# Genomic landscape of the emerging XDR *Salmonella Typhi* for mining druggable targets clpP, hisH, folP and gpmI and screening of novel TCM inhibitors, molecular docking and simulation analyses

**DOI:** 10.1186/s12866-023-02756-6

**Published:** 2023-01-21

**Authors:** Muneeba Afzal, Syed Shah Hassan, Saman Sohail, Ihosvany Camps, Yasmin Khan, Zarrin Basharat, Asad Karim, Muhammad Aurongzeb, Muhammad Irfan, Muhammad Salman, Carlos M. Morel

**Affiliations:** 1 Department of Health and Biological Sciences, Abasyn University Peshawar, Peshawar, KP 25000 Pakistan; 2 Dr. Panjwani Center for Molecular Medicine and Drug Research, International Center for Chemical and Biological Sciences, University of Karachi, Karachi, 75270 Pakistan; 3 Centre for Technological Development in Health (CDTS), Oswaldo Cruz Foundation (Fiocruz), Building “Expansão”, 8th floor room 814, Av. Brasil 4036 - Manguinhos, Rio de Janeiro, RJ 21040-361 Brazil; 4 Jamil-Ur-Rehman Center for Genome Research, PCMD-ICCBS, University of Karachi, Karachi, Sindh 75270 Pakistan; 5 Department of Chemistry, Islamia College Peshawar, Peshawar, KP 25000 Pakistan; 6 Laboratório de Modelagem Computacional, LaModel, Instituto de Ciências Exatas - ICEx. Universidade Federal de Alfenas - UNIFAL-MG, Alfenas, Minas Gerais Brazil; 7 High Performance & Quantum Computing Labs, Waterloo, Canada

**Keywords:** *Salmonella Typhi*, Subtractive genomics, Screening and ADMET profiling, MD simulation

## Abstract

Typhoid fever is transmitted by ingestion of polluted water, contaminated food, and stool of typhoid-infected individuals, mostly in developing countries with poor hygienic environments. To find novel therapeutic targets and inhibitors, We employed a subtractive genomics strategy towards *Salmonella Typhi* and the complete genomes of eight strains were primarily subjected to the EDGAR tool to predict the core genome (*n* = 3207). Human non-homology (*n* = 2450) was followed by essential genes identification (*n* = 37). The STRING database predicted maximum protein-protein interactions, followed by cellular localization. The virulent/immunogenic ability of predicted genes were checked to differentiate drug and vaccine targets. Furthermore, the 3D models of the identified putative proteins encoded by the respective genes were constructed and subjected to druggability analyses where only “highly druggable” proteins were selected for molecular docking and simulation analyses. The putative targets ATP-dependent CLP protease proteolytic subunit, Imidazole glycerol phosphate synthase hisH, 7,8-dihydropteroate synthase folP and 2,3-bisphosphoglycerate-independent phosphoglycerate mutase gpmI were screened against a drug-like library (*n* = 12,000) and top hits were selected based on H-bonds, RMSD and energy scores. Finally, the ADMET properties for novel inhibitors ZINC19340748, ZINC09319798, ZINC00494142, ZINC32918650 were optimized followed by binding free energy (MM/PBSA) calculation for ligand-receptor complexes. The findings of this work are expected to aid in expediting the identification of novel protein targets and inhibitors in combating typhoid Salmonellosis, in addition to the already existing therapies.

**Supplementary Information:**

The online version contains supplementary material available at 10.1186/s12866-023-02756-6.

## Introduction

*Salmonella Typhi* is a Gram-negative bacterium and the etiological agent of typhoid fever in humans, whereas, *Salmonella Paratyphi* A, B, and C cause a paratyphoid fever indistinguishable in clinical symptoms. The term enteric fever is used for both, i.e., typhoid *Salmonella* is referred to as *Salmonella Typhi* and *Salmonella Paratyphi* [1]. *Salmonella Typhi* subsp. *enterica* comprises more than 2600 serovars, of which four are of major medical relevance to humans. Both typhoid serovars (Typhi and Paratyphi A) are restricted to humans causing enteric disease while non-typhoidal *Salmonella* serovars (Enteritidis and Typhimurium) have a broad host range and predominantly cause gastroenteritis [2, 3]. It is still the most widespread and hazardous infection globally, especially in developing countries, where approximately 200,000 fatalities and 16 million further cases per anum have been reported [4, 5]. The main reservoir of both typhoid *Salmonella* serovars are humans, mostly observed in children. Food, contaminated water, waste, and infected individuals are the main source of transferring the organisms. Enteric fever is recognized by an incubation phase with prodromal symptoms such as headache, abdominal pain, and diarrhea (constipation) for a period of 1 week or more, followed by fever [6], whereby immunocompromised patients mostly develop constipation [7]. During infection, *Salmonella Typhi* enters epithelial cells of the small intestine and later goes through the bloodstream to infect several organs like liver, bone marrow, lymph nodes and spleen, later on re-enter the bloodstream and show fever symptoms [8].

During the early infection course, a specific fever is displayed (> 37.5 °C - 38.2 °C) followed by a gradual high fever (38.2 °C - 41.5 °C) [9]. Besides fever, bradycordia, splenomegaly, myalgia, and hepatomegaly are developed together with spots appearing on their chest and abdomen [10]. Persisting in the host cell is crucial for bacterial pathogenesis, and *Salmonella* strains possess this ability, whereas non-virulent strains fail to stay [11]. The host cell encases the bacteria in a membrane compartment and activates the immune response, thus degrading the intra-cellular bacteria via the digestive enzyme secretion and lysosomal fusion. Meanwhile, the *Salmonella* type-III secretion system injects effector proteins into the vacuole to enter the reticuloendothelial system to stay alive and proliferate [12].

Recently, the development of antimicrobial resistance (AMR) with foodborne pathogens, including *Salmonella*, has been associated with increased mortalities in humans, prolonged hospitalization, and cost/treatment factors due to therapy failure. In the 1990s and 2000s, several clones of multi-drug resistance (MDR) *Salmonella* have emerged, and their prevalence in human hosts, domestic animals, and wildlife species expanded globally [13, 14], though some antibiotics like trimethoprim-sulfamethoxazole, ampicillin, and ciprofloxacin showed good results [15]. Vaccines are one of the most effective interventions to recover public health, yet the generation of highly effective vaccines for various diseases, including salmonellosis remained hard. An important progress in the recent past is the data expansion of numerous pathogen’s genomes, proteomes, and transcriptomes. These datasets establish a groundwork for developing and employing novel methodologies to mine, and classify target proteins for the development of vaccines, drugs, and diagnostic tests. For instance, reverse vaccinology is the screening of the entire pathogen genomic data using bioinformatics tools to find antigenic outer membrane proteins as good vaccine targets followed by synthetic production and screening in infected animal models. It was first used for vaccine development against serogroup *B. meningococcal* and later, this methodology was employed against other bacteria.

Similar correlated methodologies like pangenomics and subtractive genomics have largely exposed so far, the potential targets in various challenging pathogenesis such as typhoid, paratyphoid fever, and others. These approaches employee the complete genome sequences of pathogens for predicting novel therapeutic targets and inhibitors [16–18]. In this current study, an integrated bioinformatics based subtractive genomics approach was designed for mining novel protein-based targets using the complete genomic/proteomic data of *Salmonella Typhi* and it is proposed that the same kind of approach could further be extended to other microbial pathogens.

## Material and methods

### Strains selection, data retrieval and phylogenetic analyses

*S. typhi* belongs to the phylum *Proteobacteria* and represents an important food and water-borne human pathogen, for which numerous genomes have already been sequenced worldwide, thus showing the importance of this pathogen. Briefly, We retrieved the genomic data information of *Salmonella Typhi*, available at the GOLD database (Genome Online Database) (http://gold.jgi.doe.gov) [19]. A total of eight strains of *Salmonella enterica* Typhi were included in this study. All strain files, including complete genomes, genes, and protein sequences, were retrieved from the National Center for Biotechnology Information (NCBI) (http://www.ncbi.nlm.nih.gov).

Phylogenetic tree construction for ancestral inference is a hypothetical chart representation and not definitive facts of evolutionary relationships among organisms. Their pattern of branching reflects how species evolved from a series of ordinary ancestors. For this purpose, the housekeeping gene/protein of 16S rRNA having maximum sequence length was selected for phylogenetic tree construction. A multi-fasta file that comprised of 16S rRNA genes from all strains was prepared and used as an input file. The phylogenetic tree was constructed in MEGA (v10) using neighbor-joining method [20, 21].

### Prediction of Core, non-host homologous and essential genome

To predict the core genome/proteome of *Salmonella enterica* Typhi, a high throughput automatic comparative genome analyses platform, the EDGAR v2.3 (Electronic Data Gathering Analysis and Retrieval) (https://edgar.computational.bio.uni-giessen.de) was used [22]. The EDGAR offers multiple novel web-based services and features and significantly simplifies the comparative genome analyses of related genomes via user-friendly interface. A single strain was randomly selected (*Salmonella enterica* Typhi CT 18) as the reference genome, the remaining seven strains were compared to the reference genome using the inherent default parameters. The core genome/proteome prediction is made based on % identity and coverage information provided in the EDGAR output files. From core genome analyses, the core file was submitted to NCBI-BLASTp (*e-value* = 0.0001*, bit score* = 100 & *identity* *≥ *35%) against the human genome for filtering non-host homologous proteins in the pathogen core genome. This step is important to avoid cross-reactivity with human homologous proteins. BLASTp works by identifying match regions among biological sequences. The program compares nucleotide or protein sequences to sequence databases (7 strains in this case) and calculates the significance of the statistical value (www.ncbi.nlm.nih.gov/BLASTp). A minimal set of genes important for vital activities of any cellular life is termed essential genes. The Database of Essential Genes (DEG v10) (www.essential.org) encompass experimentally validated essential genes, among others, from a number of bacterial, eukaryotic as well as archaeal species that can be comparatively used to identify essential genes in a target bacterium, e.g., of Salmonella enterica Typhi [23]. For the identification of essential genes in our target bacteria, the set of core-conserved and non-host homologous proteins from the previous step was subjected to the DEG database. The cut-off values used for BLASTp were: e­value = 0.0001, bit score ≥ 100, identity ≥ 35%, using the same parameters adapted previously [16, 17]. 

### Modelome construction through comparative homology modelling

The pool of core essential non-host homologous (CENHH) was subjected to the MHOLline server for protein 3D (three-dimensional) structure modelling (http://www.mholline.lncc.br/http://www.mholline2.lncc.br) [24]. Usually, MHOLline provides very good results, but in some cases, if the structures obtained are not of the required quality, a number of other 3D structure modeling software could be used. It predicts 3D structures for a small (a single protein sequence) as well as a large number sequences (≥ 50), hence, sometimes compromising the quality of the predicted 3D structures. The MHOLline assign group 2 (G2) to all sequences for which models can be generated, and then further classifies them into seven distinct quality groups. Sequences from very high, high, good and medium to good groups were considered where the selection of good quality structures was based on Ramachandran plot (≥ 92%). Alternatively, we deployed SWISS-MODEL (www.swissmodel.expasy.org), a fully automated online server predicting 3D model for a single target sequence using multiple template structures from the PDB database. SWISS-MODEL employs the same comparative homology modeling approach as the MHOLline server. The quality of each target was checked using structure quality validation tools including the model quality assessment at SWISS-MODEL, PDBsum available at EMBL-EBI (https://www.ebi.ac.uk/thornton-srv/databases/pdbsum/Generate.html), Verify 3D [25] and were then visualized using the PyMOL tool (http://pymol.org). Both platforms use MODELLER program but since the SWISS-MODEL predict the 3D structure for a single protein in contrary to the MHOLline workflow, it might explain the quality difference in predicted structures.

### Protein-protein interaction (PPI), cellular localization and virulence analyses

The proteins are in a homogenous environment inside the cell, performing multiple biological processes. The filtered proteins from the previous step were analyzed for protein-protein interaction (ppi) network using the STRING (v10.5) database (https://string-db.org/) [26]. *Salmonella enterica* CT18 was selected as the reference organism using the following thresholds; Network Type: full STRING network, Required score: medium confidence (0.400), FDR stringency: medium (5%). This step showed that the filtered targets were involved in multiple reactions in which the nodes stood for the selected proteins and the edges marked the interactions among the targets. The cello2go software (cello.life.nctu.edu.tw/cello2g) was next used for subcellular localizations of the final set of sequences (four .faa sequences) having 3D modeled structures [27]. The parameters used were; Blast search = bacteria, Prediction model for bacteria = gram negative, *e-value = 0.001*. The acquired results are displayed online as pie charts allowing the user to visualize the cellular localization of final targets. Finally, the molecular weight of target proteins was determined using an online bioinformatics program (www.bioinformatics.org). Furthermore, the Virulence Factor Database (VFDB, (www.mgc.ac.cn) was used to check virulence properties by recognizing epitope regions (cut-off values, *bit score* > *100*, *e-value = 0.001*, *identity* > *35%*) [28].

### ZINC library screening, molecular docking and ADMET profiling

The druggability of a protein or druggable protein pockets defines the maximum affinity of a drug-like molecule to interact with that protein. Therefore prior to druggability analysis, the DoGSiteScorer (www.DogSite.zbh.uni-hamburg.de) was used to check the availability of druggable pockets in the 3D structures of the final target proteins [29]. Virtual screening was performed by first retrieving a ligand library from the ZINC database (http://ZINC15.docking.org) [30], containing 12,000 druglike molecules, with the Tanimoto cut-off level of 60%. The template structures of all target proteins were checked for the presence of inhibitors and, where present, were used for ligand structure-based virtual screening by selecting and comparing the already predicted protein druggable cavities. In contrast, when no ligand was found in the template structure, only the druggable cavities of the target proteins were used. Later, all the protein 3D structures were checked for structural errors such as missing atoms or erroneous bonds and protonation states in the standalone MOE software (Molecular Operating Environment-v2016) following a slightly modified protocol adapted by Hassan et al., and Basharat et al.*,* [16, 31–33]. Among the top 10 hits that had the most negative scores and were able to pass Lipinski’s drug-like test were selected as suitable inhibitors. ADME/Tox analysis was performed on top-scored compounds using an ADMET prediction server (http://lmmd.ecust.edu.cn/admetsar2) to validate their parameters as suitable drug/binding candidates. Skin permeation and other physicochemical values were calculated from Swiss ADME (http://www.swissadme.ch/). Prior to docking, the structure of ligands was optimized by calculating charges, structure correction if required, applying force field (MMFF94x) and, minimizing energy. The cavities predicted via DogSiteScorer (druggability ≥0.60–0.80) for all protein targets, were compared with the cavities detected by MOE and were followed.

### Molecular dynamic simulation

The first two best complexes from docking studies were used as inputs for molecular dynamics simulations using the NAMD package v2.14 GPU [34] using the CHARMM36m force field [35–37]. The particle mesh Ewald (PME) method evaluated long-range Coulombic interactions. The integration time step was set to 2 fs. The production simulations were performed in the NPT ensemble (constant number of particles, pressure, and temperature) (*p* = 1.01325 bar and T = 300 K), using the Langevin dynamics. The solution builder module was used to generate the system topology on a cubic box with a padding of 15 Å in each direction. The TIP3P water was used to solvate the box, and Na^+^ and Cl^−^ ions, corresponding to a physiological concentration of 150 mM, were placed in the simulation box to set the ionic strength and neutralize the systems. The number of water molecules were automatically set by the solution builder module depending on the system size and ran between 12,324 and 43,602. After 10,000 steps (20 ps) of minimization, the complexes were equilibrated for 135,000 steps (270 ps). The production simulations last 200 ns. The trajectories from MD were analyzed using MD Analysis software [38, 39]. Interactions were calculated with PLIP v2.1.6 software [40].

### Binding free energy calculations by molecular mechanics Poisson Boltzmann surface area (MM/PBSA)

**Fig. 1:**
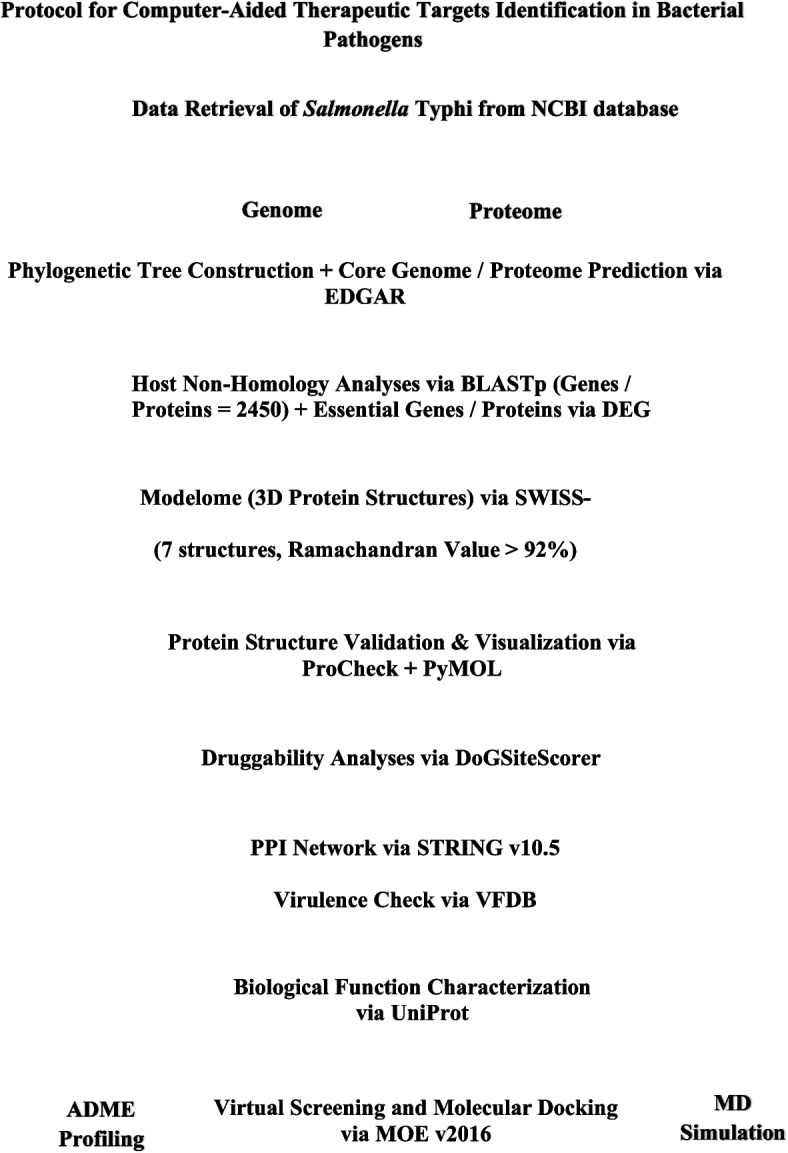
Workflow based on subtractive genomics approach describing various steps involved in protein 3D-based novel therapeutic targets identification (modified from Hassan et al.*,* 2014 [16])

The MM/PBSA method is one of the most widely adopted approaches for calculating binding free energies (ΔG_bind_) of ligands bound to biomolecule receptors after molecular docking or molecular dynamics. These calculations are performed in three steps, Molecular Mechanics (MM), Poisson Boltzmann (PB) (or generalized Born (GB), and Surface Area solvation (SA) before the summation is used to estimate the binding energy [41]. Binding free energy calculations were done using the molecular mechanics Poisson-Boltzmann surface area methodology (MM/PBSA) [42], as implemented in the CaFE package [43], a plugin of VMD software [44]. The different steps followed through subtractive genomics from data retrieval to the identification of putative protein targets are given in Fig. 1.

## Results and discussions

###  Data retrieval of selected *Salmonella Typhi* genomes / strains

The genomic data was retrieved in fasta format (.faa and .fna files) for some important *Salmonella Typhi* strains included in this study, available at the GOLD database (Genome Online Database, http://gold.jgi.doe.gov) and strains comprising their complete genomes, gene and protein sequences were retrieved from (NCBI) National center for biotechnology information (http://www.ncbi.nlm.nih.gov). This database provides a comprehensive open-source access to information regarding genome and meta-genome sequencing projects and their associated meta-data around the world. A total of eight (8) *Salmonella enterica subsp. enterica serovar* Typhi strains were included in this study. Genome statistics like genome size, number of proteins, % GC content, bio-project information and genome assembly data, among others, of all the selected strains are tabulated below (Table 1).

**Table 1:** Genome statistics of *Salmonella Typhi* strains available at National Center for Biotechnology Information (NCBI)

S. No.	Selected strains	Strains	Status	Bio- project	Assembly	Replicon	Genes	Proteins
1	***S. enterica subsp. enterica serovar*** **Typhi** ***str. CT18***	**CT18**	**Complete**	**SAMEA1705914**	**GCA_000195995.1**	**Chr 1: NC_003198.1/AL513382.1** **Pls2: (NC_003384.1/AL513383.1)**	**4829**	**4473**
2	*S. enterica subsp. enterica serovar* Typhi *str. Ty2*	**Ty2**	Complete	SAMN02604095	GCA_000007545.1	Chr1: NC_004631.1/AE014613.1	4969	4804
3	*S. enterica subsp. enterica serovar* Typhi *str. Ty21a*	**Ty21a**	Complete	PRJNA34855	GCA_000385905.1	Chr1: NC_021176.1/CP002099.1	4970	4593
4	*S. enterica subsp. enterica serovar* Typhi *str. P-stx-12*	**P-stx-12**	Complete	PRJNA80939	GCA_000245535.1	Chr1: NC_016832.1/CP003278.1 Pls: NC_016825.1/CP003279.1	5160	4473
5	*S. enterica subsp. enterica serovar* Parayphi *A str. AKU_12601*	**AKU_12601**	Complete	PRJEA30943	GCA_000026565.1	Chr1: NC_011147.1/FM200053.1	4675	4318
6	*S. enterica subsp. enterica serovar* Typhi *str. B_SF_13_03_195*	**B_SF_13_03_195**	Complete	PRJNA286162	GCA_001302625.1	**Chr1:** NZ_CP012151.1/CP012151.1	4795	4310
7	*S. enterica subsp. enterica serovar* Paratyphi *C str. RKS4594*	**RKS4594**	Complete	PRJNA20993	GCA_000018385.1	**Chr1:** NC_012125.1/CP000857.1 Pls: pSPCV:NC_012124.1/CP000858.1	4764	4414
8	*S. enterica subsp. enterica* serovar Typhi str. *BL6006*	**BL6006**	Complete	–	–	**–**	–	–

### Phylogenetic analyses

**Fig. 2:**
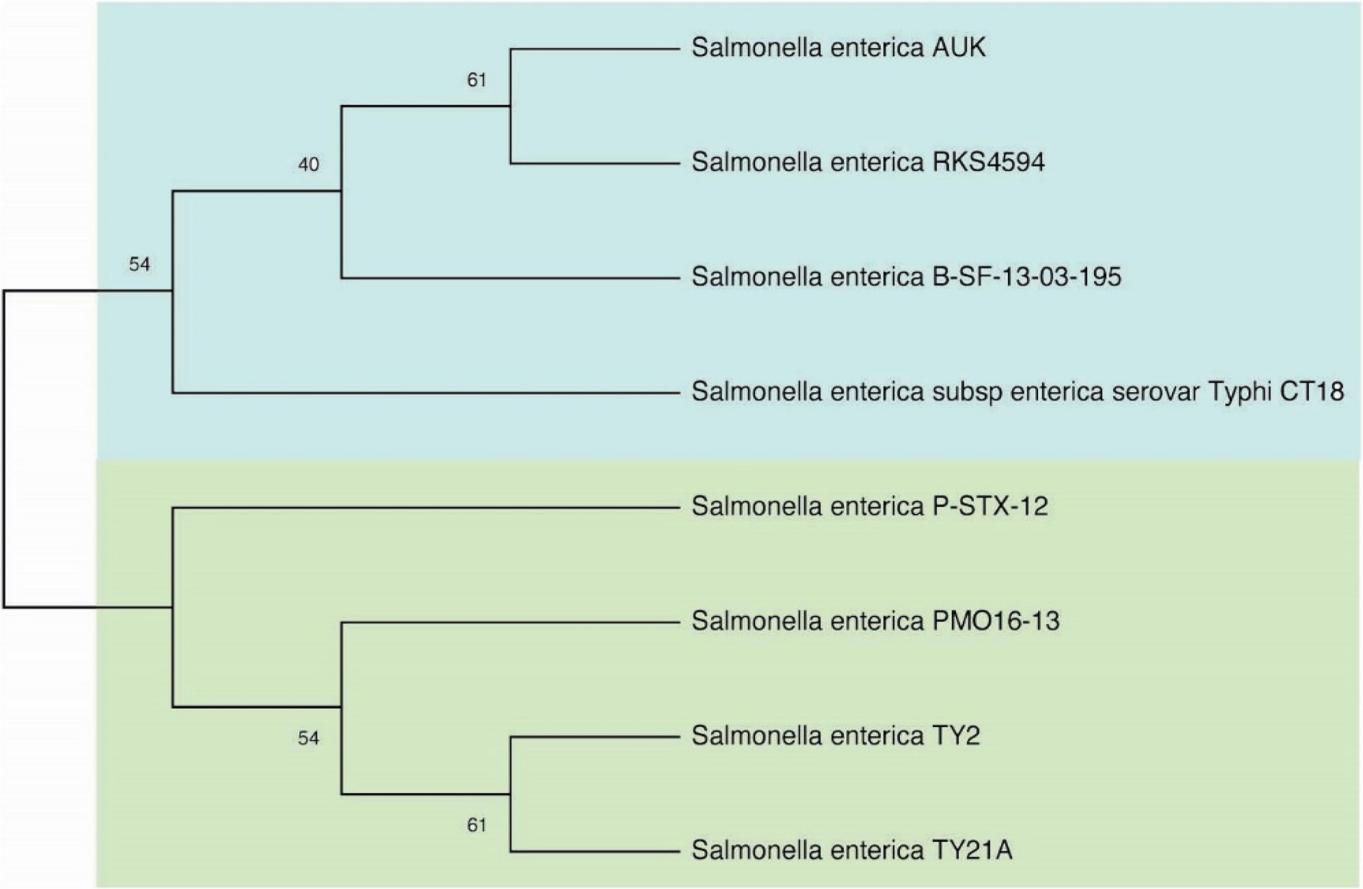
Evolutionary relationships of taxa: The evolutionary history was inferred using the Neighbor-Joining method [49] for this unrooted tree. The bootstrap consensus tree inferred from 1000 replicates is taken, with two main clusters, to represent the evolutionary history of the taxa analyzed. Branches corresponding to partitions reproduced in less than 50% bootstrap replicates are collapsed. The percentage of replicate trees in which the associated taxa clustered together in the bootstrap test (1000 replicates) are shown next to the branches. The evolutionary distances were computed using the Poisson correction method and are in the units of the number of amino acid substitutions per site [50]. This analysis involved 8 amino acid sequences. All ambiguous positions were removed for each sequence pair (pairwise deletion option). There was a total of 479 positions in the final datasets. Evolutionary analyses were conducted in MEGA (v10) [48]

A phylogenetic tree is an estimation of the relationships among taxa or sequences and their hypothetical common ancestors [45–48]. Today most phylogenetic trees are built from molecular data like DNA or protein sequences. Building a phylogenetic tree requires four distinct steps, which are as follows; step-1: identify and acquire a set of homologous DNA or protein sequences, step-2: align those sequences, step-3: estimate a tree from the aligned sequences, and step-4: present that tree in such a way as to clearly convey the relevant information to others [48]. For this purpose, we selected the long chain of 16S rRNA house-keeping genes for phylogenetic tree construction. A multi-fasta file (sequences of 16S rRNA genes from 8 strains) was prepared and used as an input file here, each constituting 479 amino acid residues. The tree was constructed in MEGA (v10) using neighbor joining method showing the relative position of each strain in comparison to others (Fig. 2).

### Mining Core genome, non-host homologous and essential genome

The core genome/proteome of *Salmonella enterica* Typhi comprised of 3207 genes/proteins. For this, *Salmonella enterica* Typhi CT18 was randomly selected as the reference genome, using the EDGAR platform with default parameters. The core region of nucleotides of selected microorganisms represents the conserved set of genes among all strains that might contain interesting therapeutics targets for drug development projects. Since *Salmonella Typhi* is a human pathogen therefore it is necessary to filter out those genes/proteins which exhibit certain degree of homology towards their host proteome, a step know as host off-targeting. The comparison to the NCBI-BLASTp program separated human homologs from the aforementioned core proteome and resulted in 2450 proteins. Afterwards, the file of 2450 proteins were submitted to the DEG database for essential genes identification. Essential genes/proteins represent a minimal set of data vital for an organism’s survival and this analysis drastically reduced our dataset to only 37 essential proteins and are given in supplementary materials (S1_table_37_targets and S1_data_37_targets).

### Modelome construction (3D comparative homology modelling)

The three-dimensional structure of proteins infers their functions and therefore are of utmost importance in understanding their role in various biological processes, specifically in pathogen target identifications projects and developing inhibitors/drugs for them. Since no experimental structural information are available in the RCSB-PDB database, therefore both MHOLline and SWISS-MODEL were deployed for protein 3D structures identification. The set of core, essential and non-host homologous (CENHH) proteins were consequently subjected to both structure prediction workflows and in total, 7 structures were obtained (S2_data_7_targets), out of which only 4 (S3_data_4_targets) showed high quality that were selected as the final targets (Ramachandran value ≥ 90%). The PDB templates identified by the SWISS-MODEL for constructing 3D models were; STY0490_WP_000122257.1=6nb1.1.A, STY2284_WP_001103591.1=4gud.1.A, STY3473_WP_000764715.1=3tzf.1.A, and STY4091_WP_000116577.1=5vpu.1.A (also Table 2). For all constructed models, the coverage between the target and template sequences was > 90%, and the identity was ≥ 50%, with the highest coverage and identity for STY0490_WP_000122257.1. In comparative homology modelling for 3D structures, these values are considered as better [16, 17, 33]. For each target, the SWISS-MODEL and the PDBsum generated the Ramachandran plots, though all the four models qualify this quality-check threshold, there is a slight variation in their Ramachandran values. In any case, a good quality 3D model would be expected to have over 90% residues in the most favored regions [1–3]. The QMEAN Z-Score demonstrates that how many standard deviations from the mean is my target model score, given a score distribution from a large set of experimentally determined structures. Thus, a Z-score around 0.0 reflect a “native-like” structure and a Z-score below − 4.0 indicates a model with low quality [51]. It is evident from Table 2 that all the four targets exhibited an acceptable QMEAN Z-Score. QMEANDisCo Global Scores are the average per-residue QMEANDisCo score, which has been found to correlate well with the lDDT score [52]. QMEANDisCo is a composite score for single model quality estimation. It employs single model scores suitable for assessing individual models, extended with a consensus component by additionally leveraging information from experimentally determined protein structures that are homologous to the model being assessed. Typically, residues showing a score below 0.6 are expected to be of low quality [53–56]. The details of different values of structure validation analyses are given in supplementary materials for all identified targets, respectively (S1_figures (a-g) – S4_figures (a-g).

**Table 2:** Molecular weight and druggability characterization of the predicted targets

Target Protein Name	Template Coverage / Identity	QMEAN Z-Scores	QMEANDisCo Scores	Ramachandran Score	Total Pockets	Highly Druggable	M. Wt (≤ 110 KDa)
STY0490 ATP-dependent CLP protease proteolytic subunit clpP	6nb1.1.A 0.95 / 99.03%	0.70	0.85 ± 0.05	94.3%	108	11	21.51KDa
STY2284 Imidazole glycerol phosphate synthase hisH	4gud.1.A 0.90 / 61.03**%**	−1.25	0.85 ± 0.06	89.9%	6	1	21.71 KDa
STY3473 7,8-dihydropteroate synthase folP	3tzf.1.A 0.98 / 74.18**%**	0.53	0.88 ± 0.05	90.3%	12	1	30.52 KDa
STY4091 2,3-bisphosphoglycerate-independent phosphoglycerate mutase gpmI	5vpu.1.A 0.99 / 62.33%	−0.78	0.87 ± 0.05	94.8%	15	2	55.56 KDa

### Molecular weight and Druggability analyses

Finally, the molecular weight of the target proteins and their respective druggable pockets/cavities were determined prior to virtual screening and molecular docking. The molecular weights (MW) of potential targets were assessed using ExPASy Server and were classified accordingly (https://web.expasy.org/compute_pi/). The druggability of a protein mlecule defines their efficiency to bind a drug-like molecule. For this purpose, the DogSiteScorer program (www.proteins.plus/www.Dogsite.zbh.uni-hamburg.de) aided in exploring the druggable pockets. The DoGSiteScorer automatically predict pockets and sub-pocket in a target protein 3D structure, performs functional characterization and druggability estimation. A highly druggable protein is considered the one that shows maximum interaction affinity toward a drug molecule. The druggability measurement is measured on a scale of 0–1, for a medium to high druggable protein, the score is ≥ 0.6 while for highly druggable protein, it is ≥ 0.8 **(**Table 2**)**. A protein of interest might contain several predicted druggable pockets yet the highly druggable pockets are normally considered for docking analyses. The drug targets were further crosschecked in the Target-Pathogen Database (http://target.sbg.qb.fcen.uba.ar) to prioritize them by determining the structural druggability, essentiality and different metabolic roles.

### Protein-protein interaction network

**Fig. 3:**
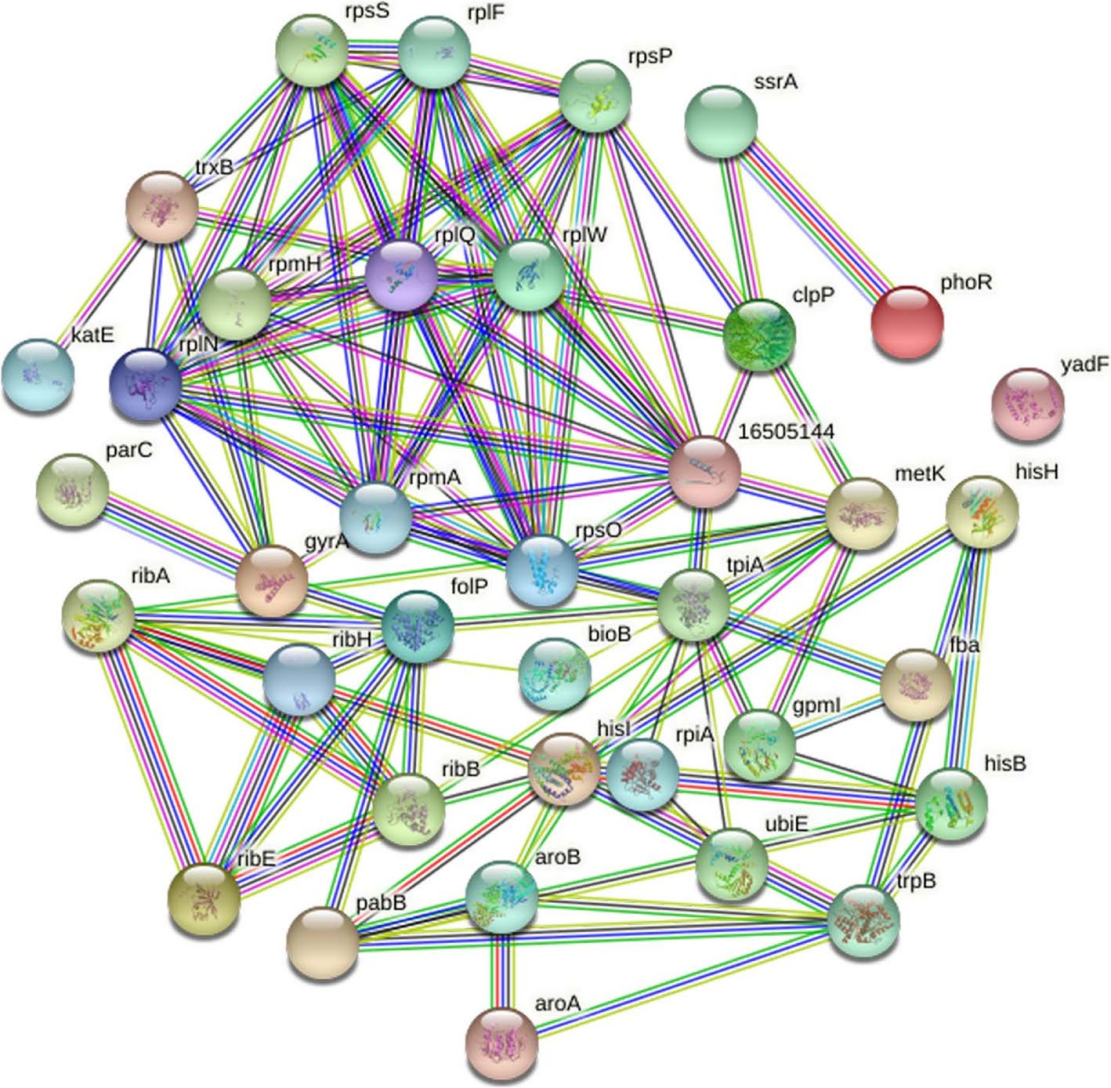
Protein-protein interaction using STRING database. The different nodes in the network represent the proteins while the network edges represent specific and meaningful protein-protein associations. The network is a scalable vector graphic [SVG]; interactive. The different node colors show the different level of interactions whereas the edge colors show their known, predicted and other interactions. The colored nodes show the query proteins and first shell of interactors, the white nodes represent second shell of interactors, empty nodes represent proteins of unknown 3D structure and filled nodes represent some 3D structure is known or predicted. The edges indicate both functional and physical protein associations whereas line color indicates the type of interaction evidence and the line thickness indicates the strength of data support. Among the known Interactions, Cyan are from curated databases and Purple are experimentally determined. In Predicted Interactions, green is from gene neighborhood analyses, red are gene fusions events, and blue are from gene co-occurrence. The other remaining interactions are; Olive = text-mining, black = co-expression, Navy Blue = protein homology

The current STRING database contains information of about 24,584,628 proteins and their interactions from more than 5000 organisms. Mainly these interactions are derived from 5 sources including a) predictions at genomic data b) high-throughput wet-lab experimental data c) co-expression data from conserved sequences d) automatic text mining from literature etc., and e) previous knowledge in other databases. It is an integrated bioinformatics web database of known (direct physical/experimental data) and indirect predicted protein–protein interactions (functional association data). The interactome for 37 essential and non-host homologs was build that was useful to check interactions of the target proteins with the neighbors. We emphasized our search whether our predicted targets were involved in more than a single interaction or not (≥ 3 interactions) explaining the promiscuous nature of the target proteins (Fig. 3). The network statistics showed the total number of nodes (*n* = 37), edges (*n* = 109) and the expected number of edges (*n* = 28). The average node degree or average number of interactions exhibited by a protein was 5.89, with average local clustering coefficient of 0.658. The PPI enrichment *p*-value (< 1.0^− 16^) was significant, the network showed significantly more interactions than it was expected. The interaction enrichment means that these proteins have more interactions among themselves than what would be expected for a random set of proteins of the same size and degree distribution drawn from the genome. Such an enrichment indicates that the proteins are at least partially biologically connected, as a group.

### Sub-cellular localization and virulence prediction

**Fig. 4:**
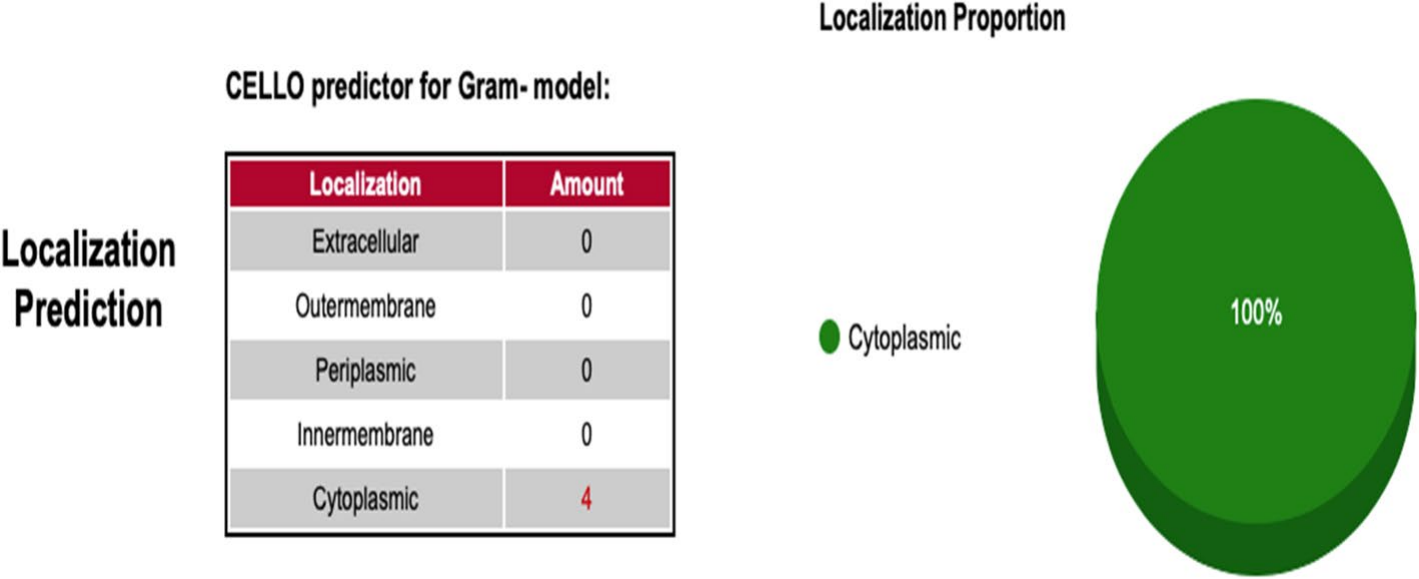
Subcellular localization of final 4 targets using CELLO2GO software. The identified putative targets were found in the cytoplasm of the *S. typhi*

A vector-based machine method and suffix tree algorithm feature, Cello2GO software investigated the subcellular location of target proteins of *S. typhi* for exo-proteome and secretome, a source of vaccine candidates due to their continuous contact with biotic and abiotic elements of the extracellular environment. It was found that all putative targets belonged to the cytoplasmic region of the pathogen cell (Fig. 4). The Virulence Factor Database (VFDB) checked the targets for virulent proteins that are involved in disease intensity, a property associated with microbial pathogenesis. This step is important because antigenic/virulent proteins could serve worthy vaccine candidates since they intervene in serious flagging pathways in the host cells and might potentially activates the host immune system in contrast to non-virulent proteins. The VFDB predicted two targets as virulent proteins (STY0490_clpP_ATP-dependent protease proteolytic subunit_WP_000122257) and (STY2284_hisH_Imidazole glycerol phosphate synthase_WP_001103591) by producing significant alignments with the VFDB core dataset proteins associated with experimentally verified 4188 sequences (virulence factors VFs). Albeit being cytoplasmic in nature, they might have an indirect role in cellular signaling or a metabolic pathway to propagate virulence and disease outcome.

### Virtual screening, molecular docking and ADMET profiling

**Fig. 5:**
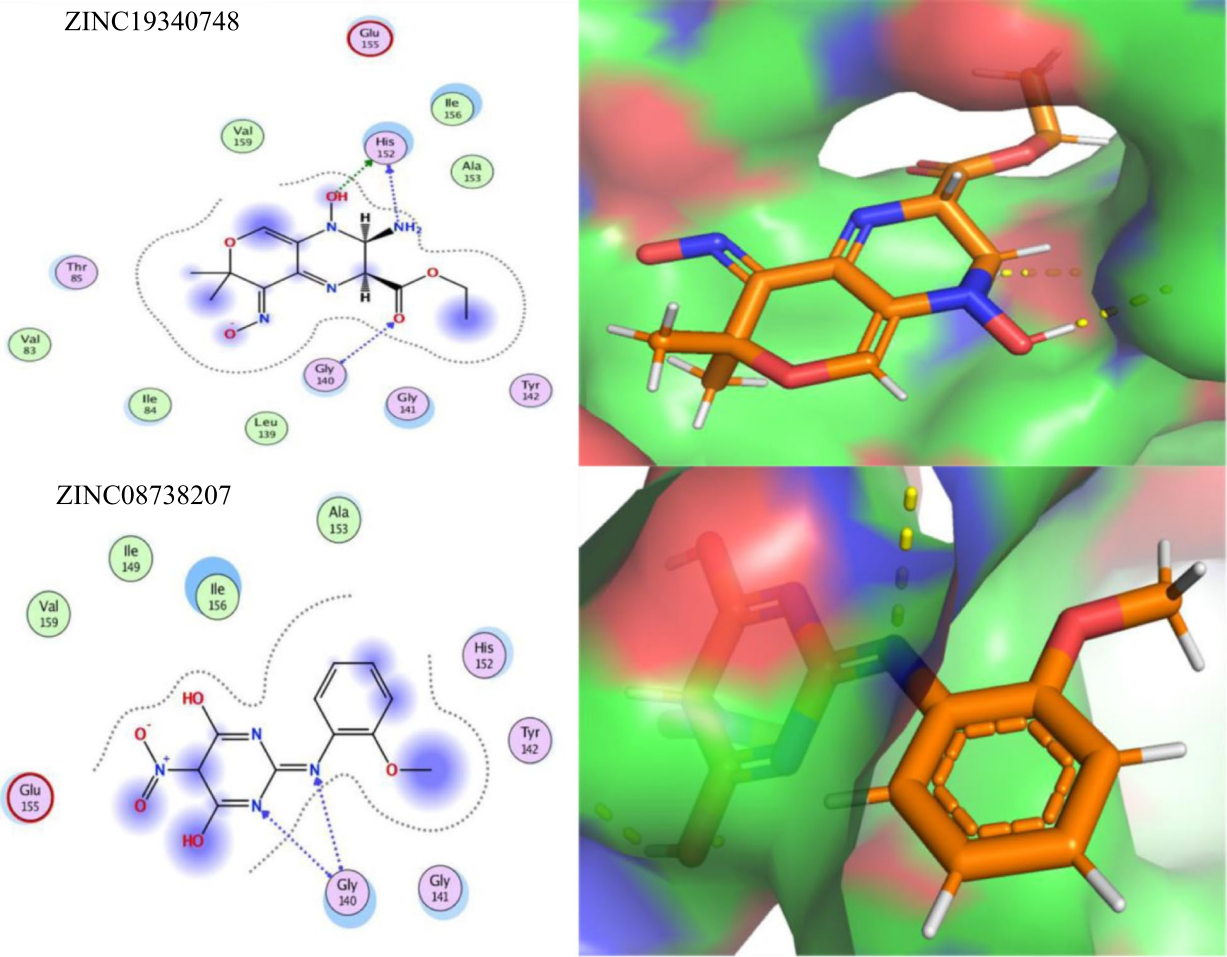
Diagram showing In Silico interactions of 2 best ZINC compounds (ZINC19340748 and ZINC08738207) with the identified putative target STY0490_ATP-dependent CLP protease proteolytic subunit. The 2D interactions (left panel) were determined via MOE software (v2016–17) while their respective 3D interactions (right panel - target protein in surface representation) were developed using PyMOL visualizing tool

**Fig. 6:**
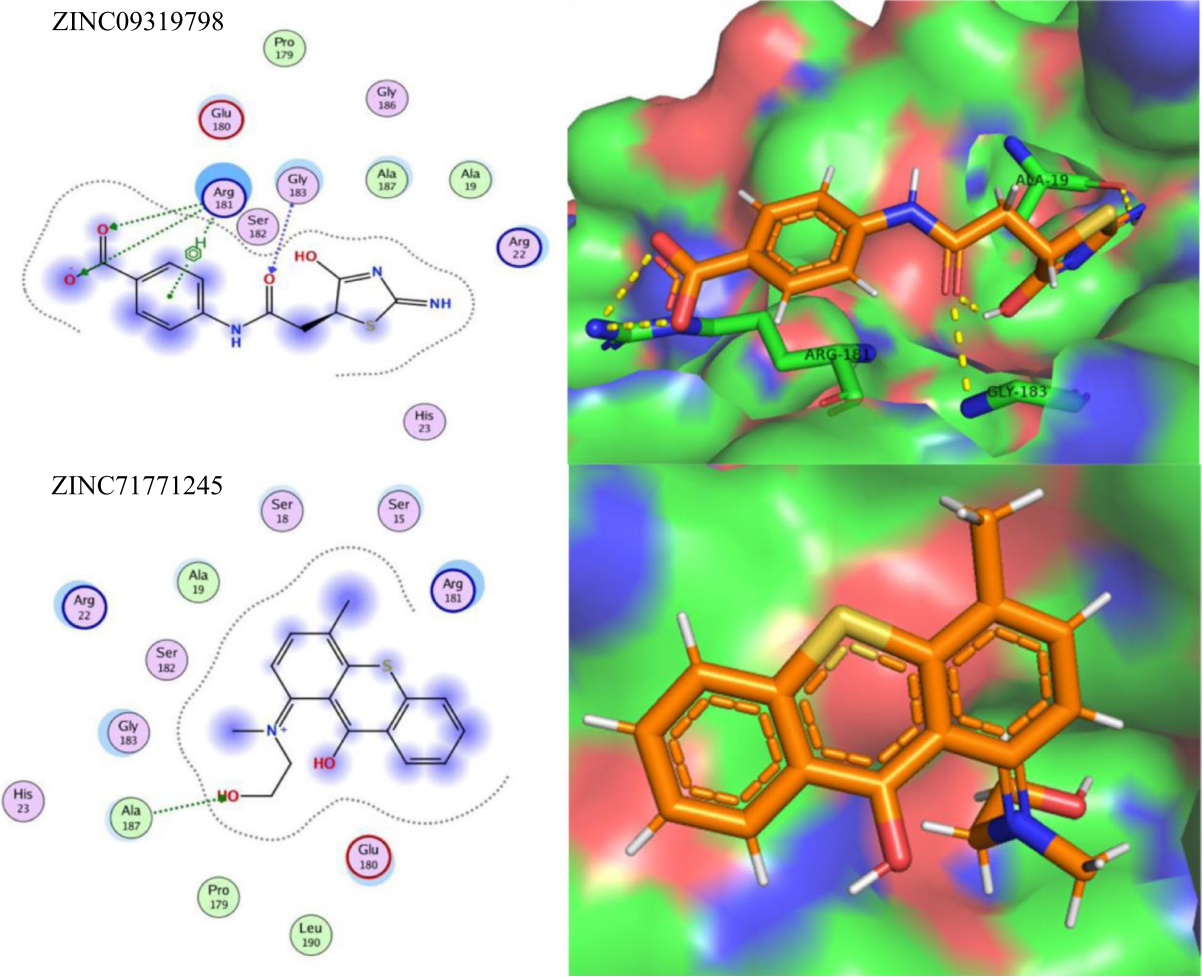
Diagram showing In Silico interactions of 2 best ZINC compounds (ZINC09319798 and ZINC71771245) with the identified putative target STY2284_hisH Imidazole glycerol phosphate synthase subunit HisH. The 2D interactions (left panel) were determined via MOE software (v2016–17) while their respective 3D interactions (right panel - target protein in surface representation) were developed using PyMOL visualizing tool

**Fig. 7:**
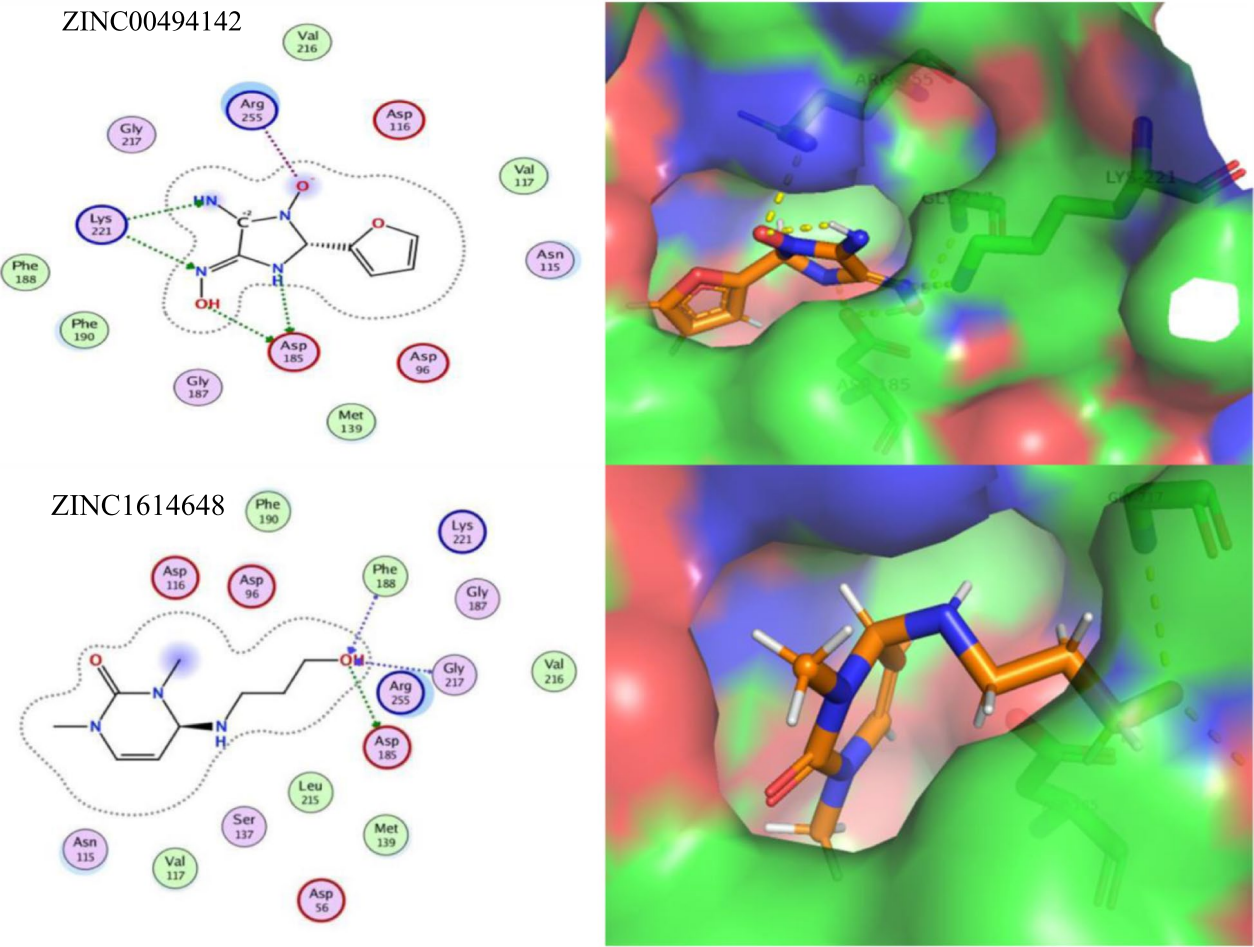
Diagram showing In Silico interactions of 2 best ZINC compounds (ZINC00494142 and ZINC1614648) with the identified putative target STY3473 Dihydropteroate synthase. The 2D interactions (left panel) were determined via MOE software (v2016–17) while their respective 3D interactions (right panel - target protein in surface representation) were developed using PyMOL visualizing tool

**Fig. 8:**
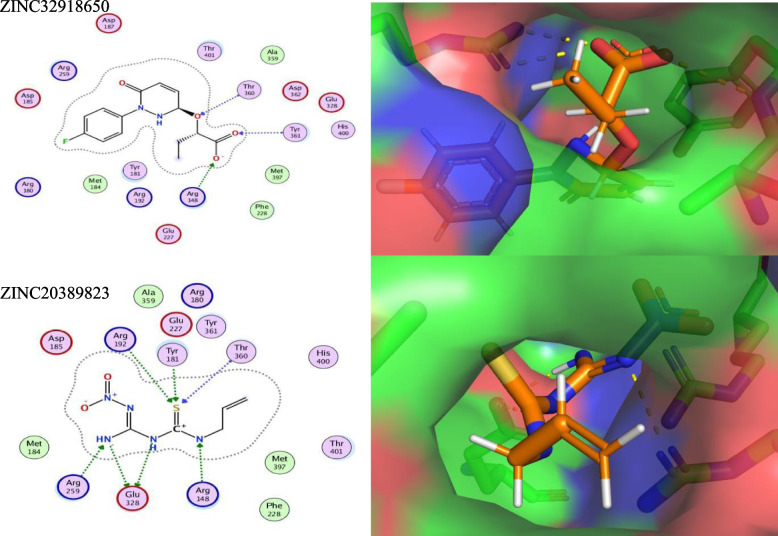
Diagram showing In Silico interactions of 2 best ZINC compounds (ZINC32918650 and ZINC20389823) with the identified putative target STY4091 2,3-bisphosphoglycerate-independent phosphoglycerate mutase. The 2D interactions (left panel) were determined via MOE software (v2016–17) while their respective 3D interactions (right panel - target protein in surface representation) were developed using PyMOL visualizing tool

After performing virtual screening, the top 200 hits were selected from the ZINC library of 12,000 molecules for each target protein (top drug-like molecules based on minimum ligand-receptor complex energy, RMSD scores and maximum number of Hydrogen bonds (H-bonding). These were then docked in the final set of our target proteins using the MOE software (15 poses selected for each ligand in the highly druggable protein cavity) and then visually inspected. In MOE, docking and visualization were performed according to a slightly modified protocol by Basharat et al., 2021; placement = triangle matcher, rescoring 1 = London dG, refinement = forcefield, rescoring 2 = affinity dG. All docked ZINC compounds were arranged in ascending order according to their binding energies and those with least energy of ligand-receptor complex were considered as top conformation. Compounds that were able to pass Lipinski’s drug-like test and had minimum energy were selected as suitable inhibitors. Later, the top 10 best drug-like molecules were selected that showed favorable interactions, favorable docking orientation and minimum energy scores for each target protein. ZINC codes and MolDock scores of selected ligands, the number of hydrogen bonds as well as protein residues involved in these interactions are tabulated **(**Tables 3, 4, 5 and 6). For convenience, the figures (Figs. 5, 6, 7 and 8) represent docking results of the top two ligands only while for MD simulation and energy calculation, only the 1st of the top two ligands was selected. In silico pharmacokinetics and pharmacology properties of selected compounds were studies for absorption distribution metabolism and excretion (ADME), to filter out the best possible drug candidate, with higher penetration and least side effects to the human and other possible hosts. Some of these compounds showed blood-brain barrier permeability or mutagenicity while most of them were substrates for P-glycoprotein. Majority of them also did not show maximum inhibition of cytochromes. Some compounds were predicted positive for mutagenicity, albeit, majority were not, in the predicted AMES toxicity test, it is presumed that they do not cause mutations in the host DNA replication or translation processes. Nearly all compounds exhibited the least acute oral toxicity for humans. Since only 2 compounds were characterized for each target protein from the top 10 hits, it is presumed that toxic compounds perilous to humans or other hosts, if any, could be replaced with the remaining 8 inhibitors from the list for ADMET profiling. Log P (o/w) is the lipophilicity of a molecule that is expressed as a partition coefficient (Log P) of an n-octanol/water system, where more lipophilic compounds are partitioned in the n-octanol layer. For a drug molecule to reach its target, it will be required to pass through lipid cell membranes, the drug requires to be sufficiently soluble in a lipid medium. For drug molecules that require oral administration, thay cannot be overly lipophilic since this will lead to poor absorption and hence will deviate the Lipinski’s ‘rule of five’ that predicts likely poor absorption or permeability when the Log *P* value is greater than five [57, 58]. The Log *K*p values, on the other hand, is another physicochemical property that show the skin permeability coefficient (*K*p) of a compound through mammalian epidermis and thus provide an insight into the mechanism of molecular transport through the stratum corneum (SC) [59]. The drug-like compounds mined in this study as potential inhibitor candidates were found to be active, safe and have not previously been studies as anti-*Salmonella* to date. These novel candidates might be interesting to be explored as *Salmonella* inhibitors, owing to future laboratory tests. The biological importance of each target and an analysis of the predicted protein-ligand interaction are described below (Table 7).

**Table 3:** STY0490**_**ATP-dependent CLP protease proteolytic subunit: Top - 10 ZINC compounds from a library of 12,000 drug-like compounds with minimum energy scores / maximum H-bond

S. No.	ZINC ID	Score (kcal/mol)	2D Interactions
1	**ZINC19340748**	**−5.8724**	**His152, Gly140.**
2	**ZINC08738207**	**−5.7044**	**Gly140**
3	ZINC83429827	−5.6663	GlyA140, ArgB132
4	ZINC08536413	−5.4598	GlyA141, IIeA156
5	ZINC16941742	−5.5048	HisA152
6	ZINC33888075	−4.7700	ArgB132
7	ZINC09319798	−5.3005	AlaC153
8	ZINC03852531	−5.2317	His152
9	ZINC00440425	−5.1016	ArgB132
10	ZINC06655690	−5.0493	GlyA140

**Table 4:** STY2284_Imidazole glycerol phosphate synthase: Top −10 ZINC compounds from a library of 12,000 drug-like compounds with minimum energy scores / maximum H-bond

S. No.	ZINC ID	Score (kcal/mol)	2D Interactions
1	**ZINC09319798**	**−5.7257**	**Arg181, Gly183**
2	**ZINC71771245**	**−5.5123**	**Ala187**
3	ZINC04876827	−5.4462	Trp118, Pro178
4	ZINC05002395	−5.2251	Val142, Arg181
5	ZINC36585021	−5.2436	Gly183
6	ZINC67743322	−5.2282	Gly183, Arg181, Tyr140
7	ZINC04521524	−5.1224	Glu180, Arg181
8	ZINC40266587	−5.0669	Tyr140
9	ZINC08655469	−5.0449	Gly48, Glu89, Val142
10	ZINC05593430	−5.0485	Gly183

**Table 5:** STY3473_Dihydropteroate synthase: Top - 10 ZINC compounds from a library of 12,000 drug-like compounds with minimum energy scores / maximum H-bond

S. No.	ZINC ID	Score (kcal/mol)	2D-Interaction
1	**ZINC00494142**	**−6.4818**	**Lys221, Asp185, Arg255.**
2	**ZINC1614648**	**− 6.2975**	**Arg255, Lys221, Gly217, Asp185, Asn115, Met139**
3	ZINC1404681	−6.2576	Asp185, Met139
4	ZINC31163220	−6.1968	Gly217, Asp185, Met139, Asn115
5	ZINC04521524	−6.0752	Asn115, Asp185, Met139, Arg255, Lys221
6	ZINC05722559	−6.0137	Arg255, Asn115
7	ZINC05468369	−5.7699	Arg255, Lys221, Gly217, Asp185, Gly187, Met139
8	ZINC05593430	−5.6062	Asp96, Gly58
9	ZINC06659051	−5.5168	Met139, Asp56, Lys221
10	ZINC67743322	−6.0257	Lys221

**Table 6:** STY4091_2,3- bisphosphoglycerate-independent phosphoglycerate mutase: Top - 10 ZINC compounds from a library of 12,000 drug-like compounds with minimum energy scores / maximum H-bond

S. No.	ZINC ID	Score (kcal/mol)	2D Interactions
1	**ZINC32918650**	**−7.3714**	**Thr360, Thr361, Arg148**
2	**ZINC20389823**	**−6.7390**	**Arg192, Tyr181, Thr360, Arg148, Glu328, Arg259**
3	ZINC71777356	−6.4355	Arg148
4	ZINC04718072	−6.2037	Arg261, Asp258, Glu328, Thy330
5	ZINC17748644	−6.1492	Arg192, Asp185, Tyr181, Tyr181, Glu227, Arg148, Arg259, Glu328
6	ZINC01582533	−6.0171	Arg192, Glu328, Asp185, Tyr181, Arg180, Arg259, Arg148, Glu227
7	ZINC68222743	−6.1124	Tyr181, Arg259, Arg148, Tyr361
8	ZINC17043741	−5.8952	Asp185, Arg259, Arg180, Arg148
9	ZINC13136442	−5.6630	Arg148, Tyr361, Thr360
10	ZINC71789643	−5.5325	Glu227, Arg180, Arg259, Arg148, Tyr181, Glu328, Arg192

**Table 7:** Pharmacokinetic parameters of the top-scoring ZINC compounds for predicted targets in *S. typhi*

Targets - *S. typhi*	Compounds	Molar refractivity	Polar surface area topology (Å^2^)	Bioavailability	Lipinski violations	Lead likeness violations	Consensus Log *P o/w*	Skin permeation Log *K*p (cm/s)
STY0490	**ZINC19340748**	79.18	129.97	0.55	0	3	−0.18	−7.94
	**ZINC08738207**	76.78	125.61	0.55	0	3	−0.48	−7.61
STY2284	**ZINC09319798**	77.53	147.15	0.55	0	0	0.42	−8.01
	**ZINC71771245**	88.40	71.71	0.55	0	0	1.96	−6.91
STY3473	**ZINC00494142**	55.39	112.53	0.55	0	1	−0.65	−7.72
	**ZINC1614648**	71.26	26.30	0.55	0	1	3.91	−4.19
STY4091	**ZINC32918650**	79.57	78.87	0.56	0	0	1.05	−8.46
	**ZINC20389823**	54.59	147.39	–	–	–	–	–

### MD simulation and binding free energy calculations by MM/PBSA

The physicochemical and thermodynamic stabilities of the four predicted targets interacting with their corresponding inhibitors, the protein-ligand complexes, depends upon several properties like the free binding energy, the number of interactions, the root mean square deviation (RMSD), the root mean square fluctuation (RMSF) and the radius of gyration (Rg). Table 8 demonstrates the free binding energies (ΔG) for each of the four complexes. All the energies have negative values which indicates a favorable protein-ligand complex formation.

**Table 8:** Free binding energy calculations of stable complexes during the last 25 ns (250 frames) of the molecular dynamic simulation (order of the increased values of the free binding energy)

Target-Inhibitor Complex ID	ΔG (kcal/mol) for 250 frames
STY0490_ ZINC19340748	−7.2039
STY4091_ ZINC32918650	−2.9975
STY2284_ ZINC09319798	−2.2178
STY3473_ ZINC00494142	−1.6335

**Fig. 9:**
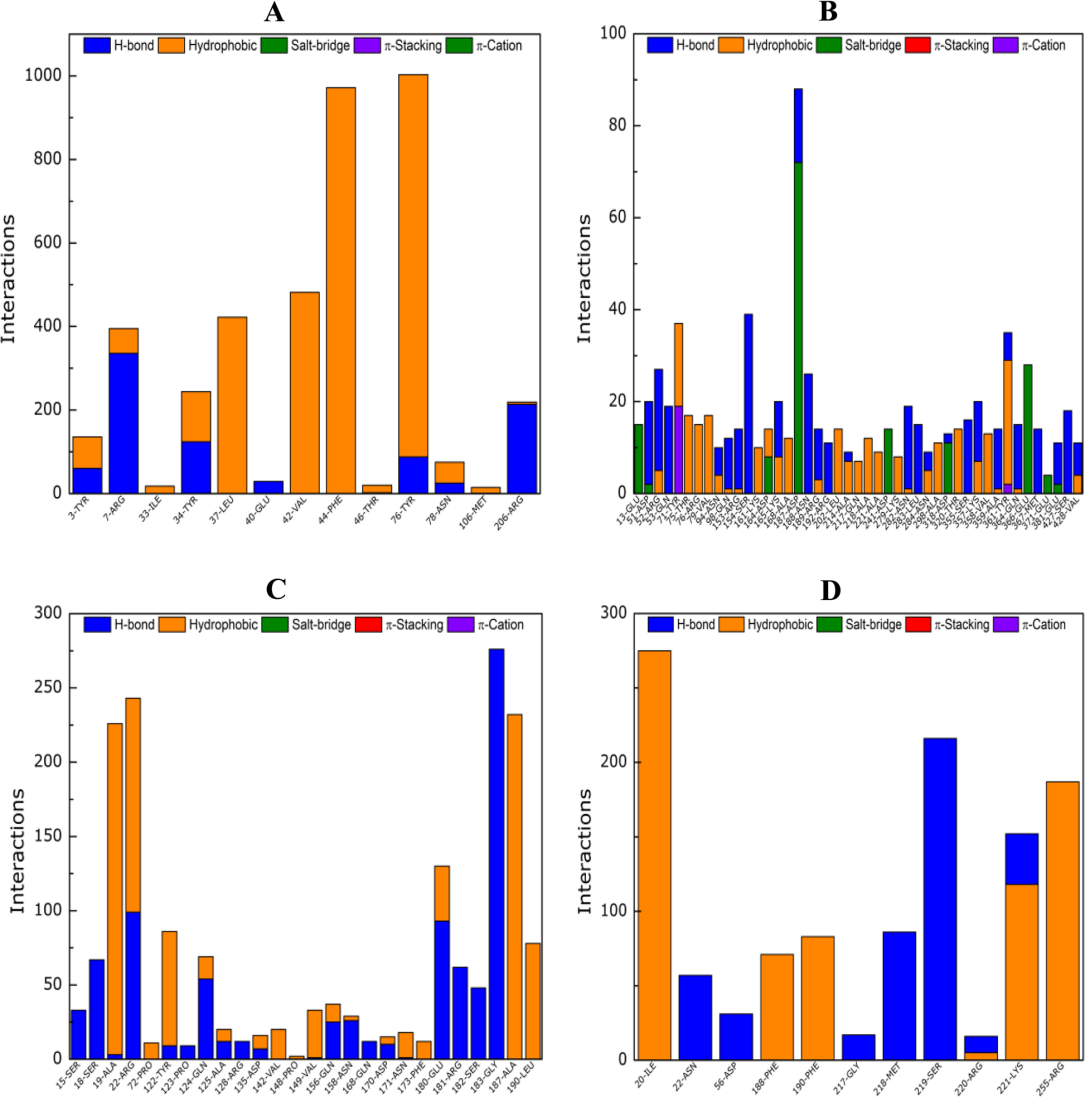
Free binding energy calculations: Interactions calculated for., **A**) STY0490_ ZINC19340748., **B**) STY4091_ ZINC32918650., **C**) STY2284_ ZINC09319798., **D**) STY3473_ ZINC00494142

Using the PLIP software, the number of hydrogen bonds (H-bond), hydrophobic contacts, salt-bridge, π-π stacking and π-cation interactions through the simulation were determined for each complex. The figures below show the calculated contacts for all the residues (only residues with more than ten (10) interactions were taken into account). The results show that the three complexes with lower free binding energy also have the greater number of interactions (greater than 500) and that the main interaction mechanisms are due to hydrophobic and H-bond contacts. The complexes with lowest number of contacts (05 and 06) show a diversity of contacts were salt-bridge, π-π stacking and π-cation also contribute to their stability (Fig. 9).

**Fig. 10:**
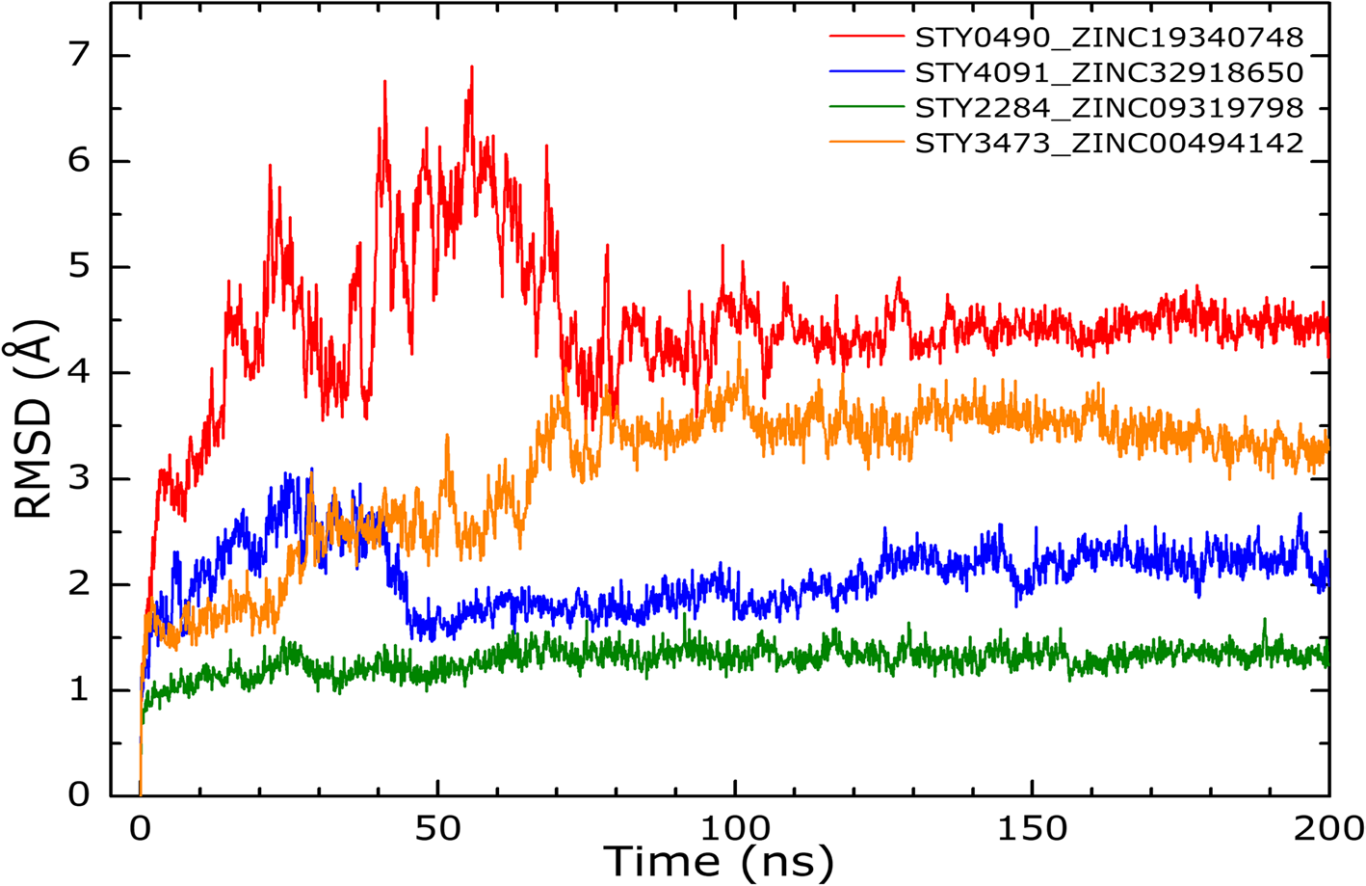
RMSD curves: The curves were calculated for., A) STY0490_ ZINC19340748., B) STY4091_ ZINC32918650., C) STY2284_ ZINC09319798 and D) STY3473_ZINC00494142

The RMSD calculated for all the complexes is shown in the Fig. 10. As can be seen, all the complexes show stability. Complexes (A and C) have some oscillations at the first half of the simulation but then attain stability after 100–125 ns whereas the other complexes (B and 06) attain stability around the first 50 ns.

**Fig. 11:**
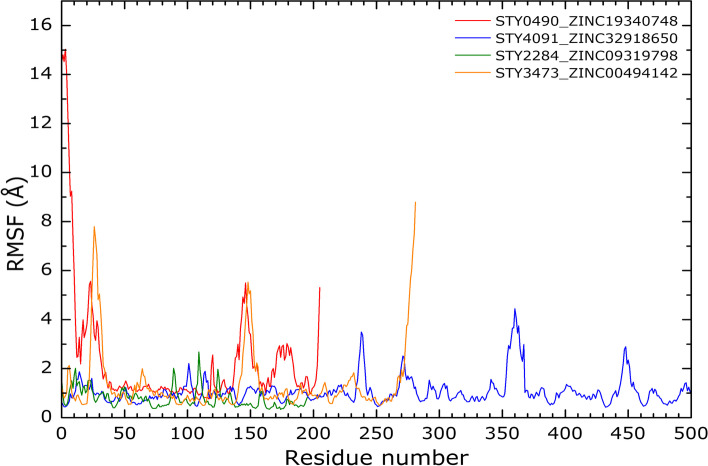
RMSF curves: The curves were calculated for., A) STY0490_ ZINC19340748., B) STY4091_ ZINC32918650., C) STY2284_ ZINC09319798 and D) STY3473_ZINC00494142

The RMSF is a measure of the residue fluctuations. Looking for the residues that made the greater number of interactions from Fig. 11, their RMSF values are lower than 4 Å. Complexes B and D, that have the greatest variety of interactions, show the lower RMSF values.

**Fig. 12:**
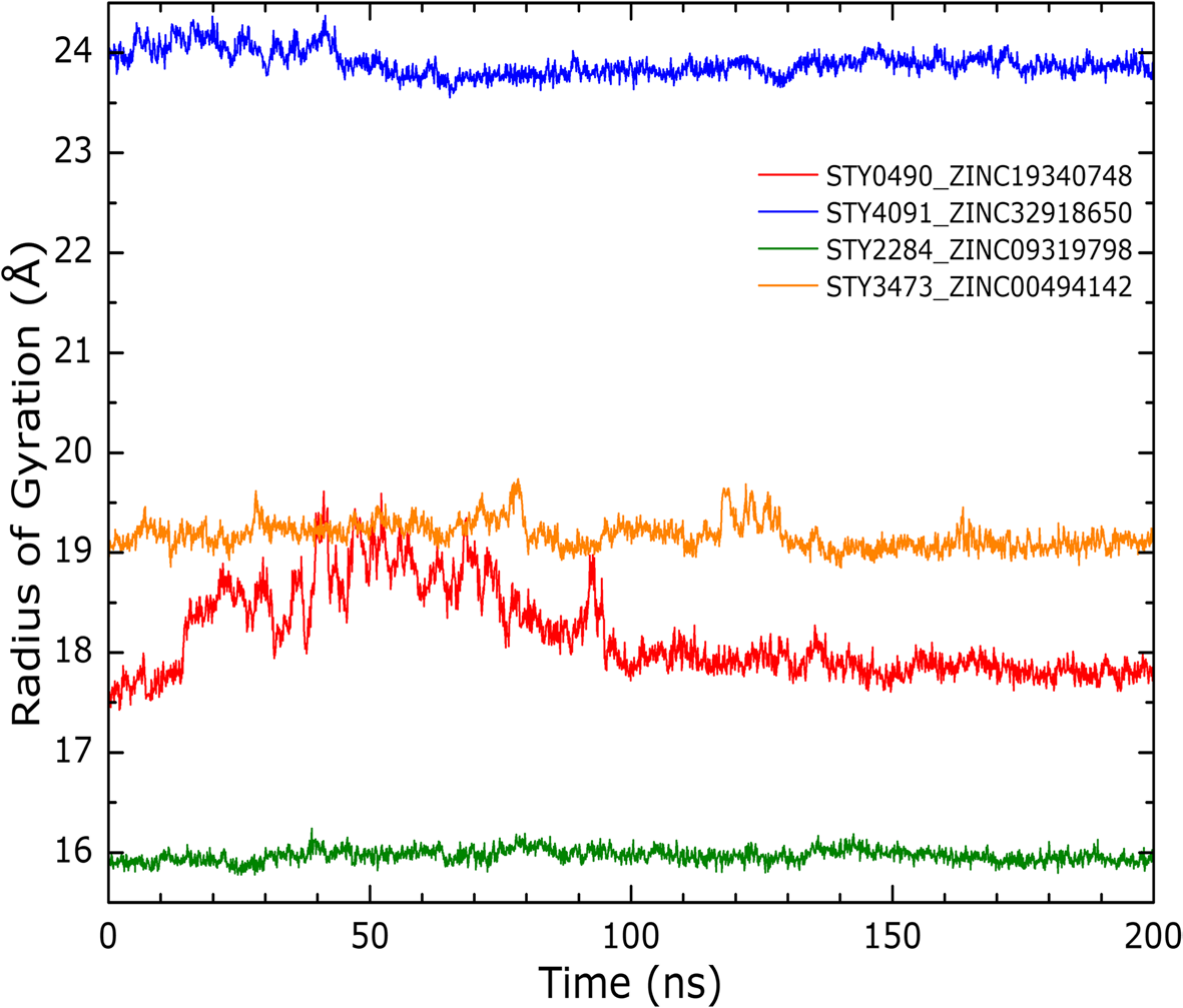
Rg curves: The curves were calculated for., A) STY0490_ ZINC19340748., B) STY4091_ ZINC32918650., C) STY2284_ ZINC09319798 and D) STY3473_ZINC00494142

The radius of gyration (Rg) can verify how compact or not the protein becomes in the complex as it measures the hydrodynamic capacity of the protein. From Fig. 12, it is observed that in all cases, the radii of gyration show a stable value with oscillations inside the 1.5 Å window.

STY 0490_clpP (EC 3.4.21.92) ATP-dependent CLP protease proteolytic subunit is a caseinolytic serine protease that cleaves peptides in various proteins that require ATP hydrolysis. ClpP has a chymotrypsin-like activity by playing a major role in the degradation of misfolded proteins. The catalytic activity comprises the hydrolysis of proteins to small peptides in the presence of ATP and Magnesium where alpha-casein is the usual test substrate, the absence of ATP causes hydrolysis of only oligopeptides shorter than five residues. It has been proved that alteration of the ClpP function is closely related to the altered virulence and infectivity of a number of pathogens thereby rendering ClpP as an attractive and potentially viable target for antivirulence drugs and antibiotics to tackle the pathogen by the activation or inhibition of ClpP [60–62]. The physiological role of the ClpP proteolytic subunit and their ability to degrade misfolded proteins generated under different stress conditions in *S. typhimurium* and other bacteria has also been reported by constructing an in-frame deletion of the clpP gene [63, 64]. The VS and docking showed 2 best hits including ZINC19340748 and ZINC08738207 that interact with the residues His152, Gly140 and Gly140, respectively in the predicted druggable pocket with the least possible ligand-receptor energy values (− 5.8724 and − 5.7044, respectively) whereas other best hits are also tabulated (Table 3 and Fig. 5).

STY2284_hisH (4.3.2.10) Imidazole glycerol phosphate synthase subunit HisH (IGPS) (CHEBI:58525). This protein is involved in step 5 of the 9-step-subpathway of L-histidine biosynthesis pathway, an Amino-acid biosynthesis pathway and synthesizes/catalyzes the conversion of PRFAR (5-[(5-phospho-1-deoxy-D-ribulos-1-ylimino) methylamino]-1-(5-phospho-β-D-ribosyl) imidazole-4-carboxamide) and glutamine (L-glutamine) to IGP (D-erythro-1-(imidazol-4-yl) glycerol 3-phosphate), AICAR (5-amino-1-(5-phospho-β-D-ribosyl) imidazole-4-carboxamide) and glutamate. The HisH subunit catalyzes the hydrolysis of glutamine to glutamate and ammonia as part of the synthesis of IGP and AICAR. The resulting ammonia molecule is channeled to the active site of HisF (https://www.uniprot.org/uniprot/P0A1R5). The enzyme has been reported as a potential target for drug and herbicide development as the histidine pathway does not occur in mammals [65–67]. We showed that Arg181, Gly183 and Ala187, among others, of the predicted druggable cavity of IGPS protein interact favorably with most of the top 10 ZINC compounds, especially the top two hits i.e., ZINC09319798 and ZINC71771245, thereby supposedly aiding in the available list of drug molecules against this enzyme (Table 4 and Fig. 6).

STY3473_folP (EC 2.5.1.15) Dihydropteroate synthase. This enzyme protein catalyzes the condensation of para-aminobenzoate (pABA) with 6-hydroxymethyl-7,8-dihydropterin diphosphate (DHPt-PP) to form 7,8-dihydropteroate (H2Pte), the immediate precursor of folate derivatives possessing the Mg^+ 2^ ion. The condensation process is involved in the step-1 subpathway of the tetrahydrofolate biosynthesis pathway. The folP gene has long been reported among sulfonamide class of drugs resistance genes and has been well studied to get an insight into the evolution of drug resistance mechanisms [68, 69]. Our docking results showed that ZINC00494142 and ZINC1614648 showed good interactions with dihydropteroate synthase with Lys221, Asp185 and Arg255, among others, with minimum energy scores (Table 5 and Fig. 7).

STY4091_gpmI (5.4.2.12) 2,3-bisphosphoglycerate-independent phosphoglycerate mutase is an important cytoplasmic enzyme involved in the sub-pathway step 3 of a 5-step glycolysis pathway to catalyze the interconversion of 2-and 3-phosphoglycerate, where Mn^+ 2^ serve as a cofactor bound to the enzyme. The phosphoglycerate mutases (PGAMs, EC 5.4.2.1) are either dependent or independent of the 2,3- bisphosphoglycerate and participate in both the glycolytic and the gluconeogenic pathways in reversible isomerization and have been reported as attractive molecular target for drug development approaches in *Trypanosoma brucei* [70, 71]. A total of 15 druggable cavities were predicted where two were highly druggable (≥ 0.8) and three were medium druggable (≥ 0.6 - ≤ 0.8) representing different degree affinity towards ligand binding. Two ZINC compounds, ZINC32918650 and ZINC20389823 were shown to interact effectively with multiple amino acid residues of the predicted highly druggable cavities (Table 6 and Fig. 8).

## Conclusion

Identification of important proteins/enzymes as interesting therapeutic targets has become possible from integrated “omics data” including genomics, transcriptomics, metabolomics and proteomics using bioinformatics and computational approaches. The scientific community is emphasizing more and more in usages of methodologies such as comparative and subtractive genomics as well as other reverse vaccinology techniques for the identification of novel drug and vaccine therapeutic targets in multiple viral, bacterial, parasitic and fungal pathogens [72, 73]. The increasing availability of bioinformatics and computational tools together with the recently sequenced complete genomes, online availability of millions of natural as well as synthetic small molecular inhibitors, and the increasing drug resistance in pathogenic microorganisms has facilitated numerous in silico studies to develop pipelines for therapeutic targets identification [74–76]. Such efforts have also prompted us to perform this study in an attempt to find novel 3D based therapeutic drug targets to cope with the pathogenesis caused by *S. typhi* species. In a nutshell, bioinformatics based comparative and subtractive genomics/structural proteomics analyses has reduced the list of final therapeutic targets in selected *S. typhi* strains in a stepwise manner. Since most of the predicted therapeutic targets are involved in critical metabolic pathways of the pathogen that regulate bacterial growth, protein biosynthesis and energy metabolism, among others, a systematic way to develop inhibitors against these targets would aid in combating the chronic onsets of typhoid fever. It is expected that the drugs investigated this way might act specifically over the pathogen thereby development of drug resistance by the pathogen and toxicity to the host might be attenuated.

## Supplementary Information

**Additional file 1.**

## Data Availability

Only public datasets were used and corollary data generated is within the manuscript and/or attached as in the “supplementary materials_*S. typhi*” uploaded and submitted with this manuscript.
