# Supplementary material for: Genomic landscape of the emerging XDR Salmonella Typhi for mining druggable targets clpP, hisH, folP and gpmI and screening of novel TCM inhibitors, molecular docking and simulation analyses
Source: BMC Microbiol. 2023 Jan 21;23:25. doi: 10.1186/s12866-023-02756-6 (PMC9860245; doi:10.1186/s12866-023-02756-6)
Supplement: Supplementary file 1 — Additional file 1. [file 12866_2023_2756_MOESM1_ESM.zip › Re_supplementary materials_S. Typhi_21-12-2022/S1_table_37_targets.docx]

**Table S1.** Core, non-host homologous and essential proteins with their accession ID’s and

| **S. No.** | **Accession ID** | **Proteins Name** |
| --- | --- | --- |
| 1 | WP_000122257.1 | ATP-dependent Clp endopeptidase proteolytic subunit ClpP [clpP] |
| 2 | WP_001103591.1 | imidazole glycerol phosphate synthase subunit HisH [hisH] |
| 3 | WP_000764715.1 | dihydropteroate synthase [folP] |
| 4 | WP_000116577.1 | 2,3-bisphosphoglycerate-independent phosphoglycerate mutase [gpmI] |
| 5 | WP_000537407.1 | thioredoxin-disulfide reductase [trxB] |
| 6 | WP_000080040.1 | bifunctional histidinol-phosphatase/imidazoleglycerol-phosphate dehydratase [hisB] |
| 7 | WP_000031748.1 | elongation factor Tu [tufA] |
| 8 | WP_000091939.1 | 50S ribosomal protein L6 [rplF] |
| 9 | WP_000613954.1 | 50S ribosomal protein L14 [rplN] |
| 10 | WP_000617546.1 | 50S ribosomal protein L23 [rplW] |
| 11 | WP_001216370.1 | 50S ribosomal protein L17 [rplQ] |
| 12 | WP_000059465.1 | 30S ribosomal protein S15 [rpsO] |
| 13 | WP_000256453.1 | 30S ribosomal protein S16 [rpsP] |
| 14 | WP_001138115.1 | 30S ribosomal protein S19 [rpsS] |
| 15 | WP_000940593.1 | 50S ribosomal protein L27 [rpmA] |
| 16 | WP_000831330.1 | 50S ribosomal protein L34 [rpmH] |
| 17 | WP_001281905.1 | DNA topoisomerase IV subunit A [parC] |
| 18 | WP_001281282.1 | DNA topoisomerase (ATP-hydrolyzing) subunit A [gyrA] |
| 19 | WP_001062140.1 | methionine adenosyltransferase [metK] |
| 20 | WP_001216342.1 | triose-phosphate isomerase [tpiA] |
| 21 | WP_000189741.1 | ribose-5-phosphate isomerase RpiA [rpiA] |
| 22 | WP_000651585.1 | carbonate dehydratase [yadF] |
| 23 | WP_000019093.1 | catalase HPII [katE] |
| 24 | WP_001050754.1 | two component system sensor kinase [ssrA] |
| 25 | WP_000893648.1 | phosphate regulon sensor histidine kinase PhoR [phoR] |
| 26 | WP_000034386.1 | class II fructose-bisphosphate aldolase [fba] |
| 27 | WP_000209485.1 | tryptophan synthase subunit beta [trpB] |
| 28 | WP_000954855.1 | bifunctional phosphoribosyl-AMP cyclohydrolase/phosphoribosyl-ATP diphosphatase [hisI] |
| 29 | WP_000439825.1 | 3-dehydroquinate synthase [aroB] |
| 30 | WP_000978456.1 | aminodeoxychorismate synthase component 1 [pabB] |
| 31 | WP_000493973.1 | riboflavin synthase [ribE] |
| 32 | WP_000445205.1 | 3-phosphoshikimate 1-carboxyvinyltransferase [aroA] |
| 33 | WP_001021372.1 | 6,7-dimethyl-8-ribityllumazine synthase [ribH] |
| 34 | WP_001176284.1 | GTP cyclohydrolase II [ribA] |
| 35 | WP_001076978.1 | 3,4-dihydroxy-2-butanone-4-phosphate synthase [ribB] |
| 36 | WP_000090724.1 | biotin synthase BioB [bioB] |
| 37 | WP_000229009.1 | bifunctional demethylmenaquinone methyltransferase/2-methoxy-6-polyprenyl-1,4-benzoquinol methylase UbiE [ubiE] |

codes.
