# Supplementary material for: Genomic landscape of the emerging XDR Salmonella Typhi for mining druggable targets clpP, hisH, folP and gpmI and screening of novel TCM inhibitors, molecular docking and simulation analyses
Source: BMC Microbiol. 2023 Jan 21;23:25. doi: 10.1186/s12866-023-02756-6 (PMC9860245; doi:10.1186/s12866-023-02756-6)
Supplement: Supplementary file 1 — Additional file 1. [file 12866_2023_2756_MOESM1_ESM.zip › Re_supplementary materials_S. Typhi_21-12-2022/S3b_ figure_STY3473_Procheck_Rama.pdf]

PROCHECK

Ramachandran Plot

c156

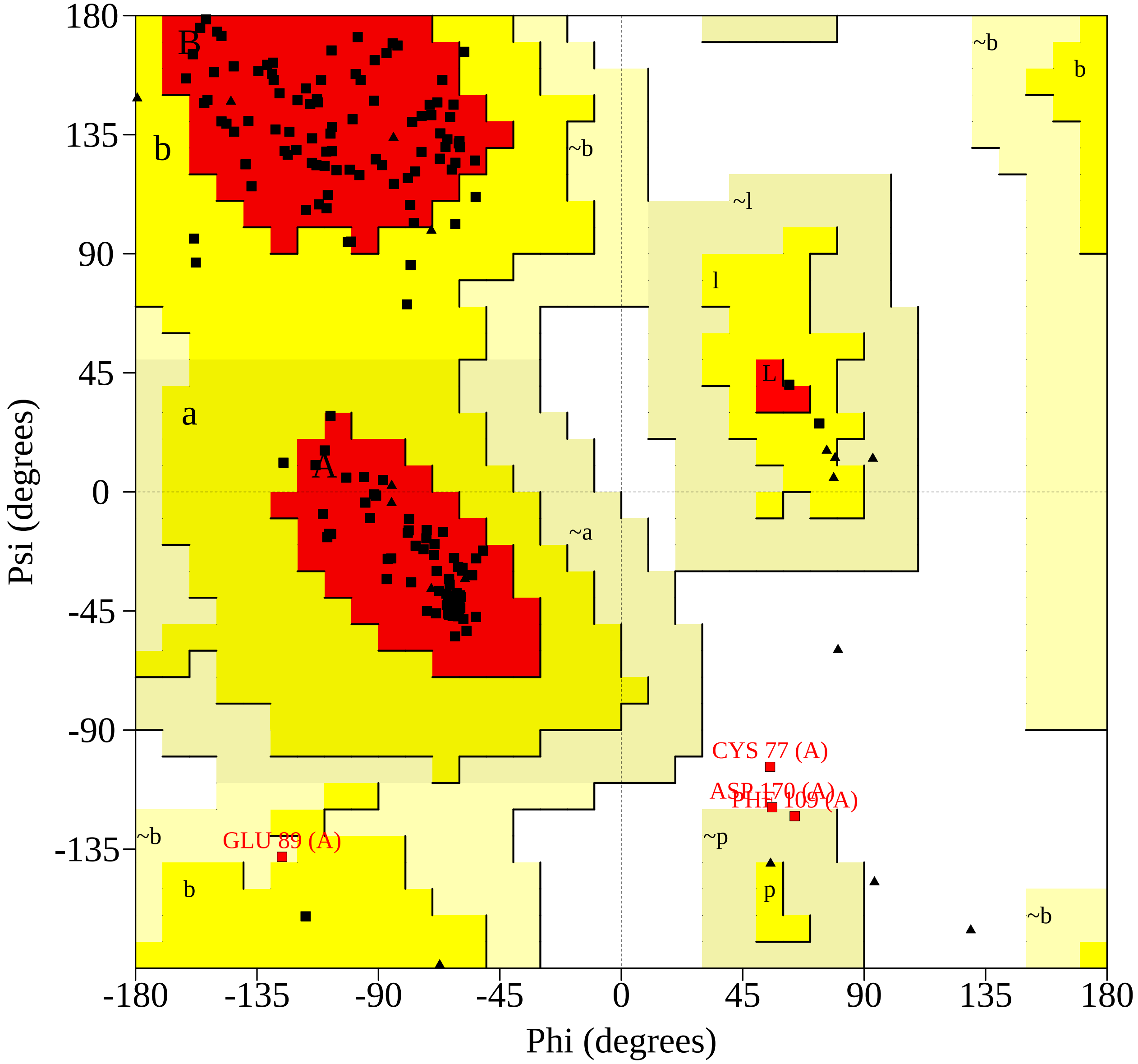

Plot statistics

Residues in most favoured regions [A,B,L]	149	90.3%
Residues in additional allowed regions [a,b,l,p]	12	7.3%
Residues in generously allowed regions [~a,~b,~l,~p]	2	1.2%
Residues in disallowed regions	2	1.2%
	----	------
Number of non-glycine and non-proline residues	165	100.0%
Number of end-residues (excl. Gly and Pro)	2	
Number of glycine residues (shown as triangles)	17	
Number of proline residues	11	

Total number of residues	195	

Based on an analysis of 118 structures of resolution of at least 2.0 Angstroms and R-factor no greater than 20%, a good quality model would be expected to have over 90% in the most favoured regions.
