# Supplementary figures and images for: Genomic landscape of the emerging XDR Salmonella Typhi for mining druggable targets clpP, hisH, folP and gpmI and screening of novel TCM inhibitors, molecular docking and simulation analyses
Source: BMC Microbiol. 2023 Jan 21;23:25. doi: 10.1186/s12866-023-02756-6 (PMC9860245; doi:10.1186/s12866-023-02756-6)

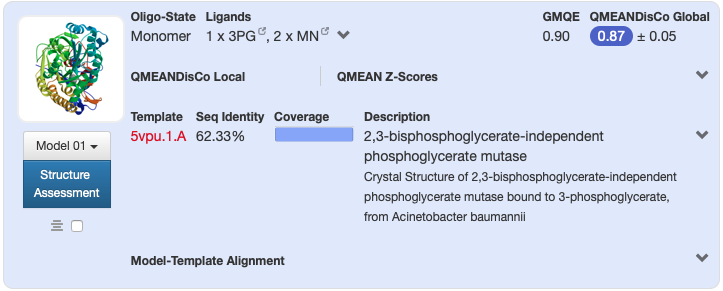

Supplement: Supplementary file 1 — Additional file 1. [file 12866_2023_2756_MOESM1_ESM.zip › Re_supplementary materials_S. Typhi_21-12-2022/S4a_ figure_STY4091.png]

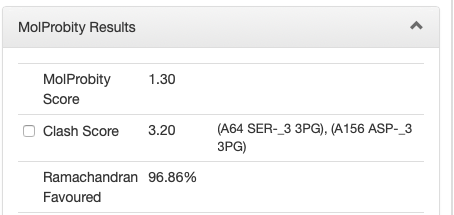

Supplement: Supplementary file 1 — Additional file 1. [file 12866_2023_2756_MOESM1_ESM.zip › Re_supplementary materials_S. Typhi_21-12-2022/S4e_ figure_STY4091.png]

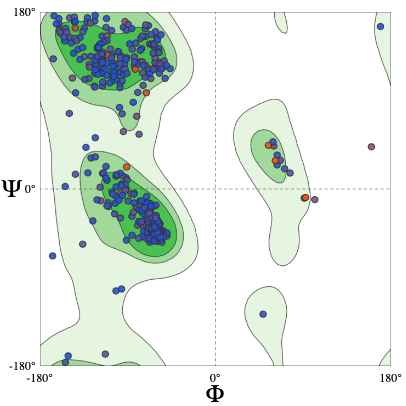

Supplement: Supplementary file 1 — Additional file 1. [file 12866_2023_2756_MOESM1_ESM.zip › Re_supplementary materials_S. Typhi_21-12-2022/S4d_ figure_STY4091.png]

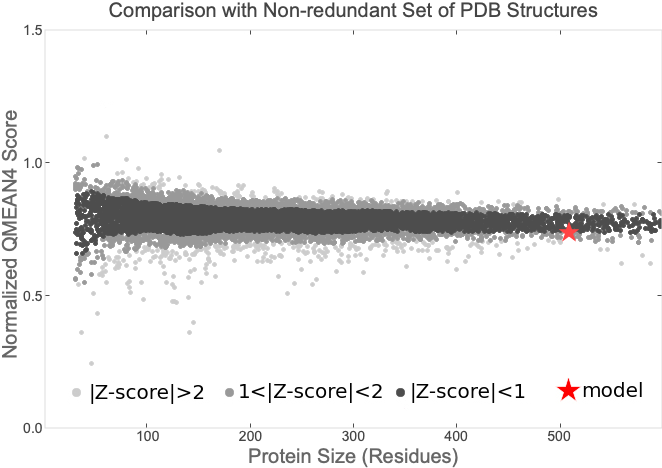

Supplement: Supplementary file 1 — Additional file 1. [file 12866_2023_2756_MOESM1_ESM.zip › Re_supplementary materials_S. Typhi_21-12-2022/S4g_ figure_STY4091.png]

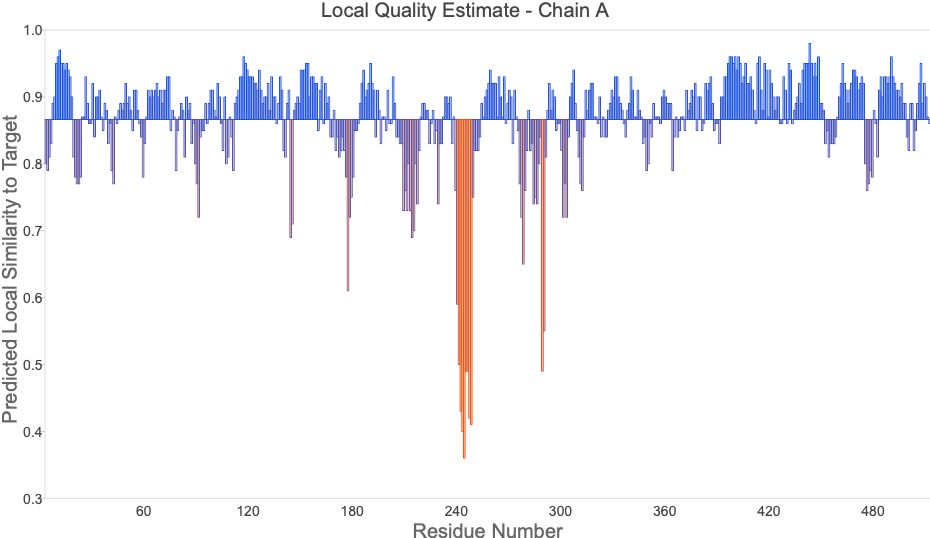

Supplement: Supplementary file 1 — Additional file 1. [file 12866_2023_2756_MOESM1_ESM.zip › Re_supplementary materials_S. Typhi_21-12-2022/S4f_ figure_STY4091.png]

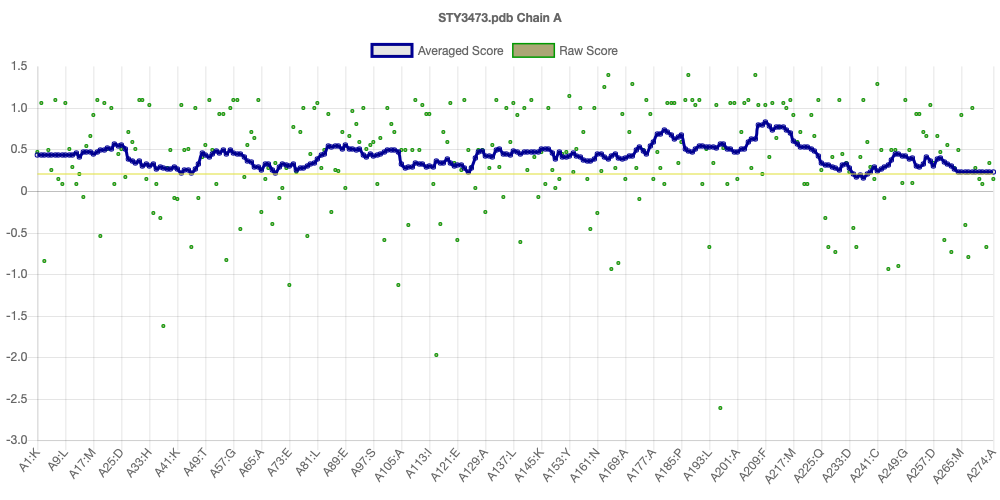

Supplement: Supplementary file 1 — Additional file 1. [file 12866_2023_2756_MOESM1_ESM.zip › Re_supplementary materials_S. Typhi_21-12-2022/S3c_ figure_STY3473_Verify3D.png]

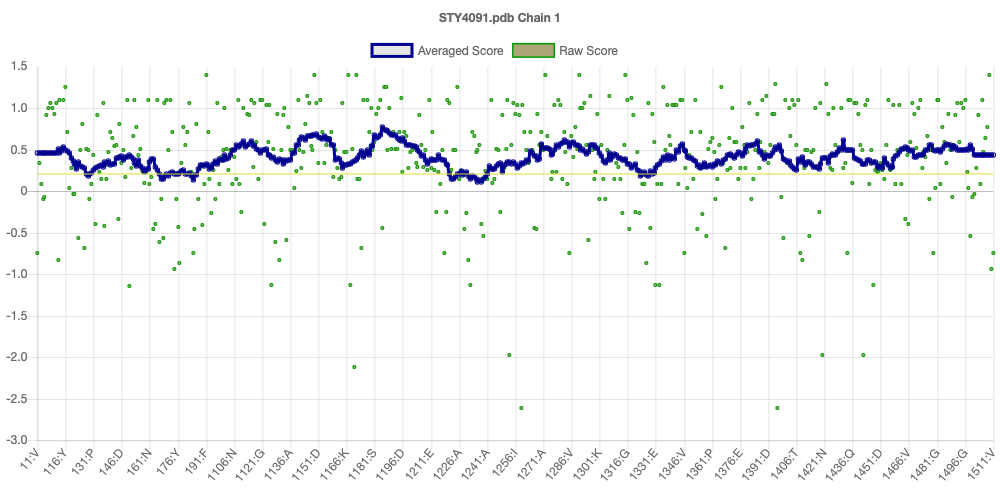

Supplement: Supplementary file 1 — Additional file 1. [file 12866_2023_2756_MOESM1_ESM.zip › Re_supplementary materials_S. Typhi_21-12-2022/S4c_ figure_STY04091_Verify3D.png]

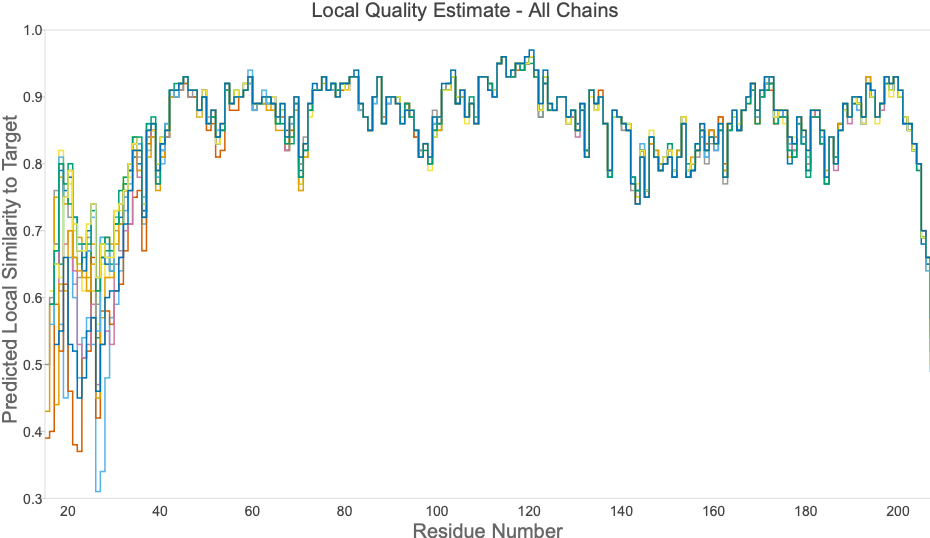

Supplement: Supplementary file 1 — Additional file 1. [file 12866_2023_2756_MOESM1_ESM.zip › Re_supplementary materials_S. Typhi_21-12-2022/S1g_ figure_STY0490.png]

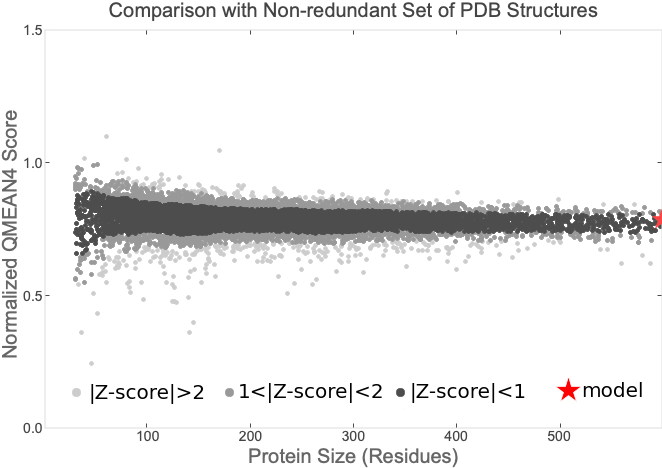

Supplement: Supplementary file 1 — Additional file 1. [file 12866_2023_2756_MOESM1_ESM.zip › Re_supplementary materials_S. Typhi_21-12-2022/S1f_ figure_STY0490.png]

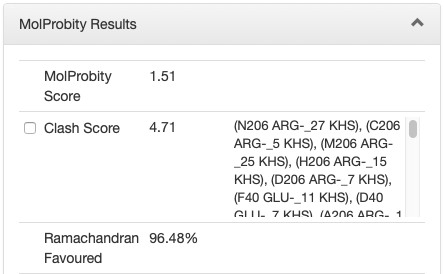

Supplement: Supplementary file 1 — Additional file 1. [file 12866_2023_2756_MOESM1_ESM.zip › Re_supplementary materials_S. Typhi_21-12-2022/S1e_ figure_STY0490.png]

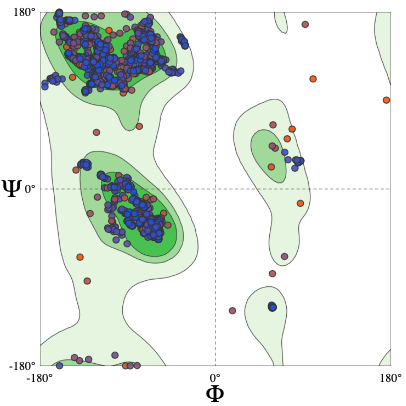

Supplement: Supplementary file 1 — Additional file 1. [file 12866_2023_2756_MOESM1_ESM.zip › Re_supplementary materials_S. Typhi_21-12-2022/S1d_ figure_STY0490.png]

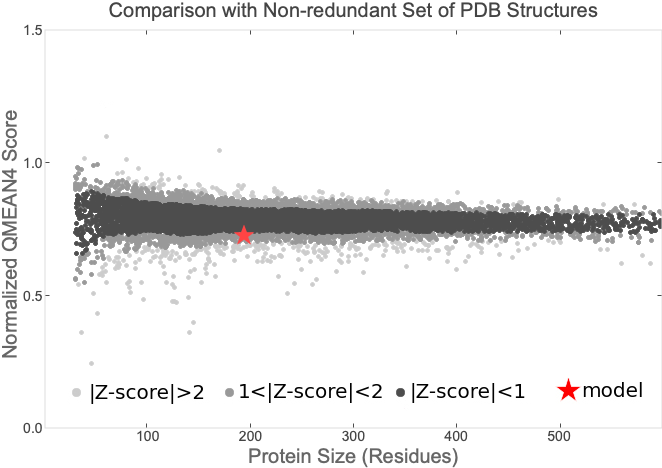

Supplement: Supplementary file 1 — Additional file 1. [file 12866_2023_2756_MOESM1_ESM.zip › Re_supplementary materials_S. Typhi_21-12-2022/S2f_ figure_STY2284.png]

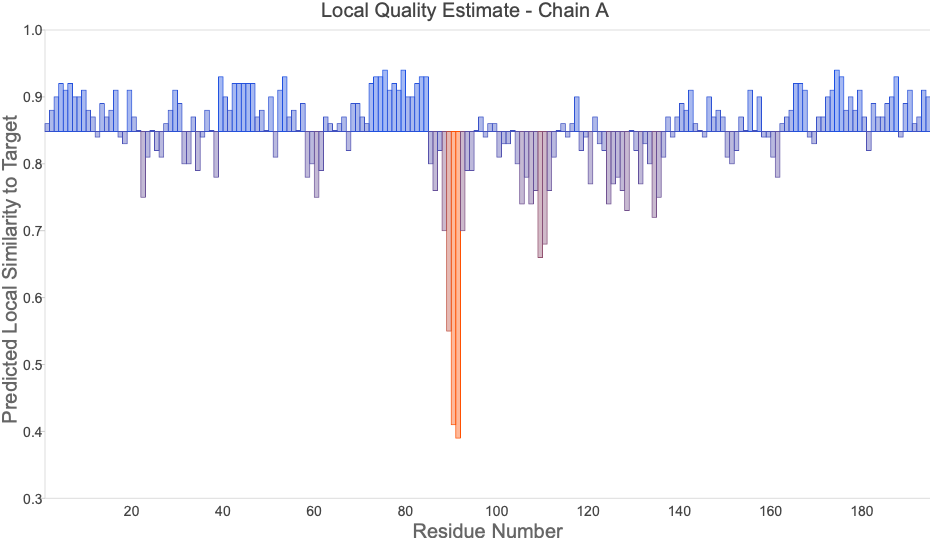

Supplement: Supplementary file 1 — Additional file 1. [file 12866_2023_2756_MOESM1_ESM.zip › Re_supplementary materials_S. Typhi_21-12-2022/S2g_ figure_STY2284.png]

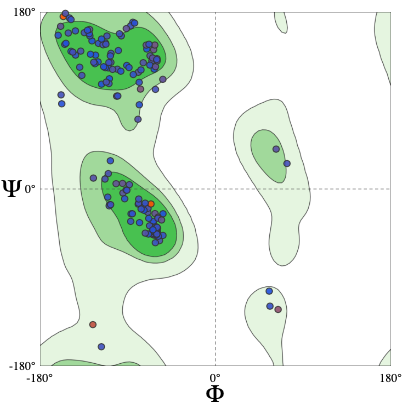

Supplement: Supplementary file 1 — Additional file 1. [file 12866_2023_2756_MOESM1_ESM.zip › Re_supplementary materials_S. Typhi_21-12-2022/S2d_ figure_STY2284.png]

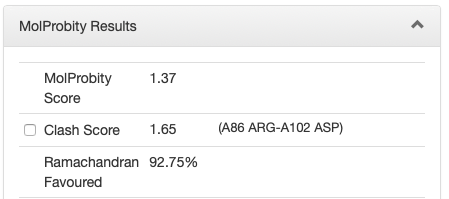

Supplement: Supplementary file 1 — Additional file 1. [file 12866_2023_2756_MOESM1_ESM.zip › Re_supplementary materials_S. Typhi_21-12-2022/S2e_ figure_STY2284.png]

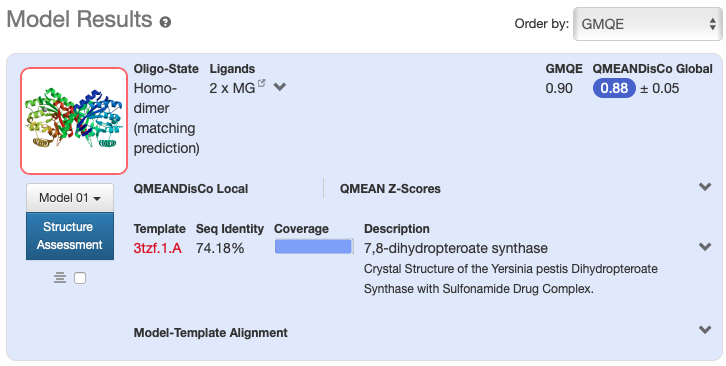

Supplement: Supplementary file 1 — Additional file 1. [file 12866_2023_2756_MOESM1_ESM.zip › Re_supplementary materials_S. Typhi_21-12-2022/S3a_ figure_STY3473 .png]

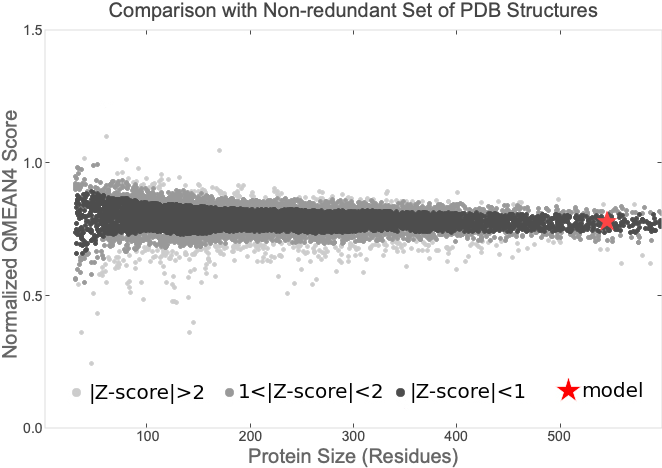

Supplement: Supplementary file 1 — Additional file 1. [file 12866_2023_2756_MOESM1_ESM.zip › Re_supplementary materials_S. Typhi_21-12-2022/S3f_ figure_STY3473.png]

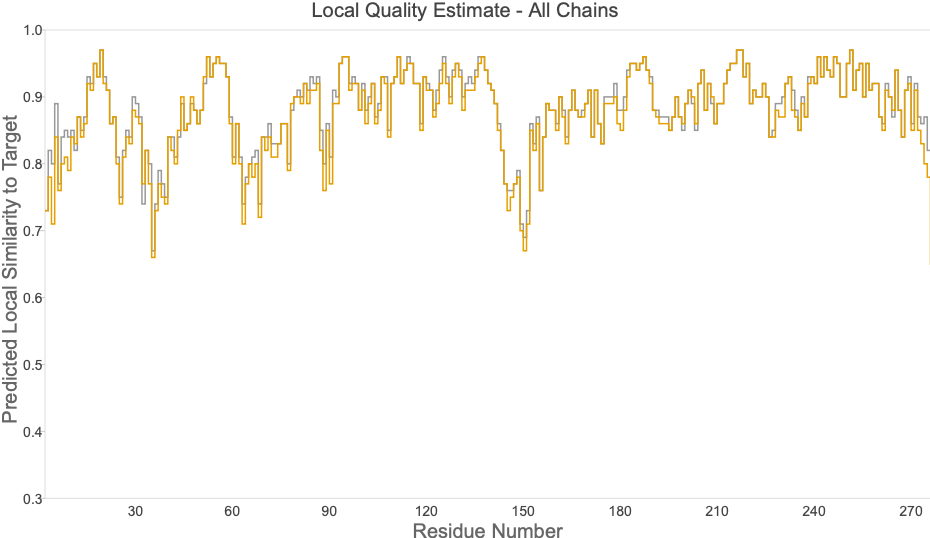

Supplement: Supplementary file 1 — Additional file 1. [file 12866_2023_2756_MOESM1_ESM.zip › Re_supplementary materials_S. Typhi_21-12-2022/S3g_ figure_STY3473.png]

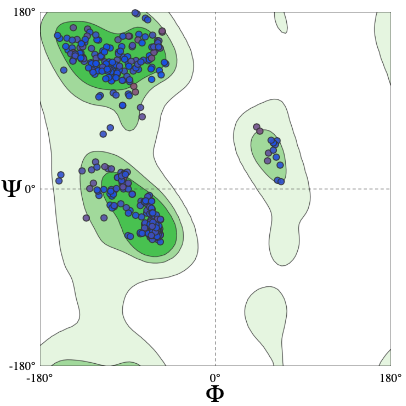

Supplement: Supplementary file 1 — Additional file 1. [file 12866_2023_2756_MOESM1_ESM.zip › Re_supplementary materials_S. Typhi_21-12-2022/S3d_ figure_STY3473.png]

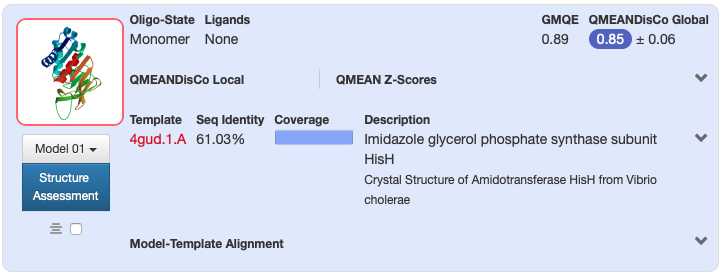

Supplement: Supplementary file 1 — Additional file 1. [file 12866_2023_2756_MOESM1_ESM.zip › Re_supplementary materials_S. Typhi_21-12-2022/S2a_ figure_STY2284.png]

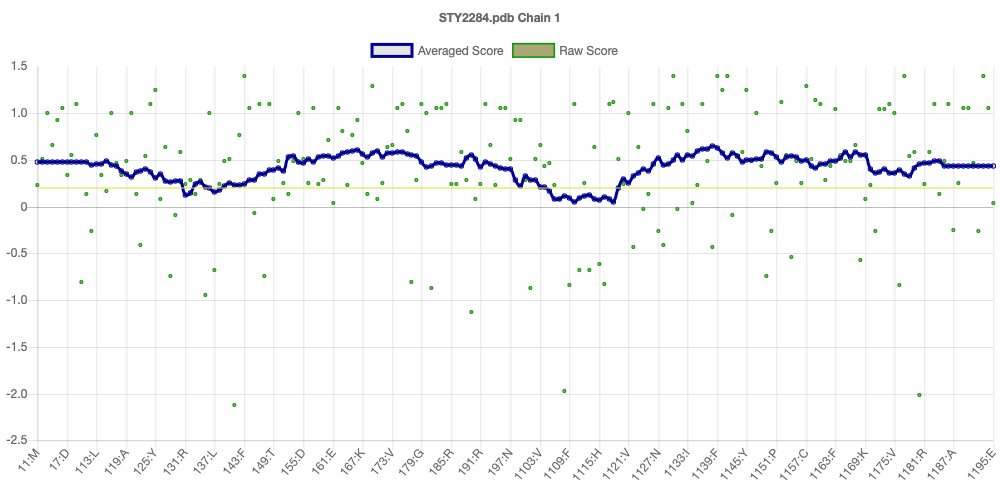

Supplement: Supplementary file 1 — Additional file 1. [file 12866_2023_2756_MOESM1_ESM.zip › Re_supplementary materials_S. Typhi_21-12-2022/S2c_ figure_STY2284_Verify3D.png]

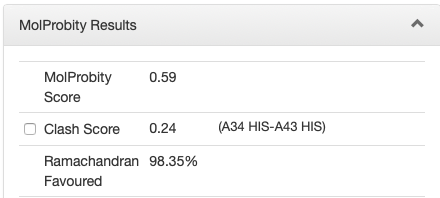

Supplement: Supplementary file 1 — Additional file 1. [file 12866_2023_2756_MOESM1_ESM.zip › Re_supplementary materials_S. Typhi_21-12-2022/S3e_ figure_STY3473.png]

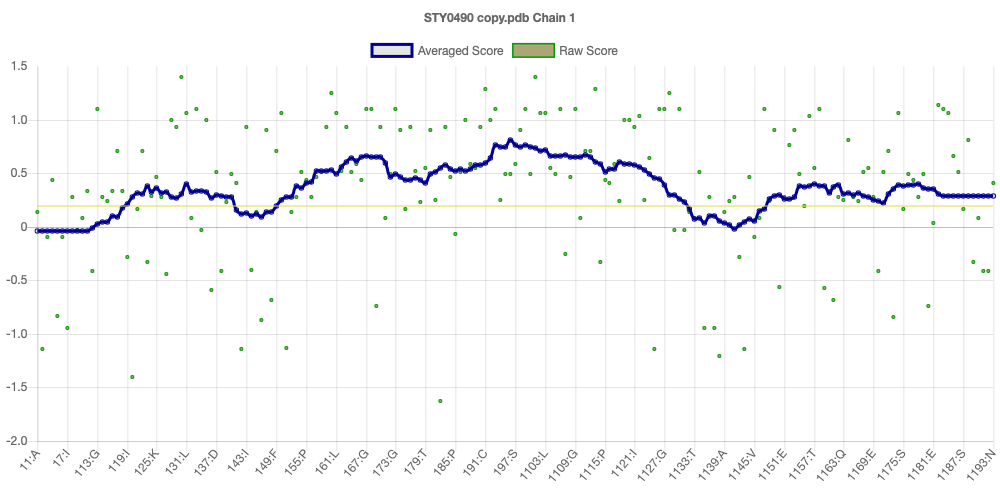

Supplement: Supplementary file 1 — Additional file 1. [file 12866_2023_2756_MOESM1_ESM.zip › Re_supplementary materials_S. Typhi_21-12-2022/S1c_ figure_STY0490_Verify3D_chain A.png]

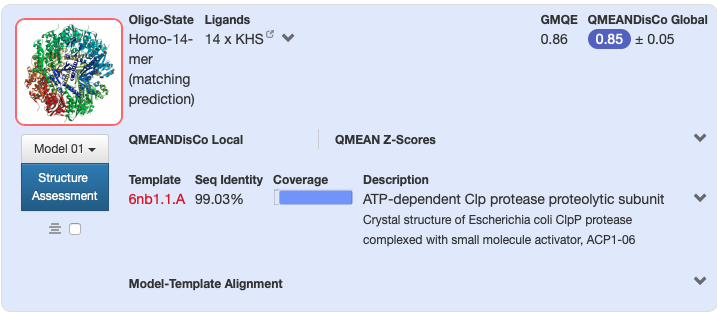

Supplement: Supplementary file 1 — Additional file 1. [file 12866_2023_2756_MOESM1_ESM.zip › Re_supplementary materials_S. Typhi_21-12-2022/S1a_ figure_STY0490 .png]
